# Notch directs telencephalic development and controls neocortical neuron fate determination by regulating microRNA levels

**DOI:** 10.1242/dev.201408

**Published:** 2023-06-05

**Authors:** Jisoo S. Han, Elizabeth Fishman-Williams, Steven C. Decker, Keiko Hino, Raenier V. Reyes, Nadean L. Brown, Sergi Simó, Anna La Torre

**Affiliations:** Department of Cell Biology and Human Anatomy, University of California Davis, Davis, CA 95616, USA

**Keywords:** Cortical development, Neurogenesis, Cell fate, Notch, miRNA, Mouse

## Abstract

The central nervous system contains a myriad of different cell types produced from multipotent neural progenitors. Neural progenitors acquire distinct cell identities depending on their spatial position, but they are also influenced by temporal cues to give rise to different cell populations over time. For instance, the progenitors of the cerebral neocortex generate different populations of excitatory projection neurons following a well-known sequence. The Notch signaling pathway plays crucial roles during this process, but the molecular mechanisms by which Notch impacts progenitor fate decisions have not been fully resolved. Here, we show that Notch signaling is essential for neocortical and hippocampal morphogenesis, and for the development of the corpus callosum and choroid plexus. Our data also indicate that, in the neocortex, Notch controls projection neuron fate determination through the regulation of two microRNA clusters that include let-7, miR-99a/100 and miR-125b. Our findings collectively suggest that balanced Notch signaling is crucial for telencephalic development and that the interplay between Notch and miRNAs is essential for the control of neocortical progenitor behaviors and neuron cell fate decisions.

## Teaser

**Summary:** Notch signaling promotes the neurogenesis of late-born cortical fates in mouse brain by upregulating the microRNAs let-7, miR-99a/100 and miR-125b.

## INTRODUCTION

The mammalian telencephalon contains an unparalleled diversity of neural populations generated during development in a tightly regulated series of events. Despite the astounding intricacies of the mature cerebrum, the telencephalon arises from a relatively simple neuroepithelial sheet composed solely of neural progenitors (Rakic, 1972; Ramón y Cajal, 1899). An exquisitely orchestrated interplay of intrinsic and extrinsic factors choreographs the emergence of distinct territories along the different axes. The posterior medio-dorsal region will develop into the hippocampus, cortical hem and choroid plexus, whereas the embryonic dorsal telencephalon will develop into the neocortex in the anterior and lateral aspects (Hebert and Fishell, 2008; Rallu et al., 2002; Wilson and Rubenstein, 2000).

At early stages of development, neural progenitors called radial glial cells (RGs) expand the whole thickness of the neocortex from the ventricular (apical) surface to the pial (basal) surface. As development proceeds and the cortex grows, the somas of the RGs remain close to lateral ventricles, forming the ventricular zone where RGs can divide symmetrically to self-renew or asymmetrically to yield intermediate progenitors and post-mitotic neurons (Sidman and Rakic, 1973; Villalba et al., 2021). Importantly, these progenitors produce excitatory projection neurons in a conserved sequential manner (Frantz and McConnell, 1996; Luskin et al., 1988; Reid et al., 1995; Walsh and Cepko, 1988). Throughout neurogenesis, newly born excitatory projection neurons use the RGs as a scaffold to migrate radially across the existing cortex and position themselves atop, forming an ‘inside-out’ lamination pattern (Noctor et al., 2004). Accordingly, the deeper neocortical layers (e.g. layer VI) are formed by early-born neurons, whereas the more superficial layers (e.g. layers II-III) contain late-born cells.

The Notch signaling pathway is a pivotal regulator of numerous developmental processes in the telencephalon, including regulating the balance between proliferation and differentiation of progenitor populations, cell fate acquisition and glial cell specification, among other roles (Artavanis-Tsakonas et al., 1999; Dorsky et al., 1995; Irvine, 1999; Jadhav et al., 2006; Yoon and Gaiano, 2005). Ligands such as Delta-like or Jagged/Serrate bind to the transmembrane Notch receptors, causing the proteolytic release of the Notch intracellular domain (NICD). NICD then translocates to the nucleus (Schroeter et al., 1998) and binds to a complex that includes RBPJ (recombination signal-binding protein for immunoglobulin κ J region, also known as CSL and CBF1), MAML1 (mastermind-like transcriptional co-activator1), p300 (EP300) and other proteins, to transcriptionally activate downstream genes (Fortini and Artavanis-Tsakonas, 1994; Smoller et al., 1990). Well-known effector targets of the Notch pathway include the HES (Hairy and Enhancer of Split) and HEY (Hairy Ears, Y-linked) families. Previous studies have reported that HES1-deficient mice exhibited accelerated neuronal differentiation in the neocortex whereas either HES1 (Ohtsuka and Kageyama, 2021) or HES5 (Bansod et al., 2017) overexpression led to an expansion of the neural progenitor pool and prolonged production of superficial layer neurons and astrocytes. Such findings suggest that the timing and levels of Notch signaling must be properly regulated to maintain the temporal control of neurogenesis.

Importantly, the Notch signaling pathway also engages in complex feedback loops with several microRNA (miRNAs) (Fishman et al., 2022). miRNAs regulate the expression of Notch pathway components, including HES1 and HES5 (Georgi and Reh, 2011; Patterson et al., 2014). At the same time, Notch activity regulates the transcription of several miRNAs in different paradigms (Roese-Koerner et al., 2016; Singh et al., 2020; Tan et al., 2012). miRNAs have recently emerged as key regulators of cortical fate acquisition and developmental timing. In particular, let-7 and miR-125b are part of the heterochronic pathway that regulates many developmental transitions in bilaterally symmetrical animals and are key components of the molecular machinery that allows neural progenitors to generate late cell populations in the cortex and retina (La Torre et al., 2013; Shu et al., 2019).

Here, we show that balanced Notch signaling is necessary for proper development of the neocortex, corpus callosum, hippocampus and choroid plexus. Additionally, we show that Notch signaling regulates neurogenesis and cortical laminar organization. At a molecular level, Notch coordinates the expression of several transcription factors, including basic helix-loop-helix (bHLH) neurogenic transcription factors as well as two miRNA clusters, *MiR99ahg* and *MiR100hg*, the host genes for the miRNAs miR-99a, let-7c and miR-125b-2; and miR-100, let-7a, and miR-125b-1, respectively. Strikingly, we demonstrate that inhibition of these miRNAs partially rescues Notch gain-of-function phenotypes *in vivo*. Together, our data indicate that complex interactions between the Notch pathway and miRNAs are essential for proper cell fate specification and overall telencephalic development.

## RESULTS

### Notch signaling regulates corpus callosum and hippocampal development

To investigate the roles of the Notch pathway during early telencephalic development, we generated Notch gain-of-function (GOF) and dominant-negative (DN) mouse transgenic lines. A GOF strain was generated by crossing ROSA26^loxP-stop-loxP-Notch1-ICD^ (Murtaugh et al., 2003) with the Emx1-Cre driver (Gorski et al., 2002). The resulting mouse line, hereafter referred to as Emx1-NICD, overexpresses Notch1-ICD in the dorsal telencephalon from embryonic day (E) 10.5. Similarly, we generated a DN line by overexpressing a truncated MAML1 protein that acts as a dominant negative (ROSA26^loxP-stop-loxP-dnMAML1^; Tu et al., 2005) using the same Emx1-Cre driver (hereafter Emx1-dnMAML). In both lines, CRE recombinase mediates excision of the loxP-flanked STOP cassette, allowing for the expression of either NICD or dnMAML. Littermates containing no CRE were used as controls for all experiments. In both cases, the constructs are inserted at the *ROSA26* locus and, thus, they are equivalent, avoiding expression differences due to the surrounding genomic DNA.

Emx1-NICD mice failed to thrive (Fig. S1A) and died at around 2 weeks of age, whereas Emx1-dnMAML mice showed no differences in animal size or survival rates compared with their control littermates. At a global level, neither of these strains show ed significant brain size differences at birth [postnatal day (P) 0], but by P10, Emx1-dnMAML animals exhibited smaller telencephalons compared with controls (Fig. S1B-D).

**Fig. 1.**
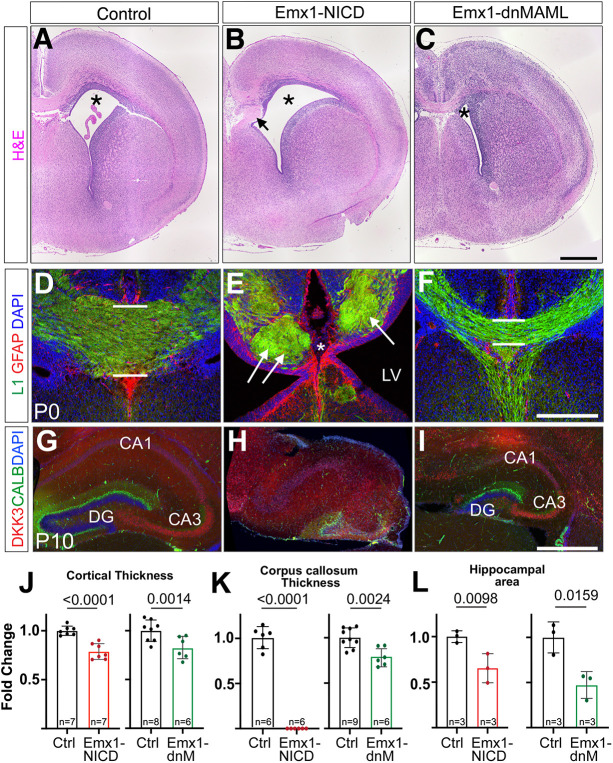
**Morphological defects in Emx1-NICD and Emx1-dnMAML models.** (A-C) Hematoxylin and eosin staining (H&E) of control (A), Emx1-NICD (B) and Emx1-dnMAML (C) P0 brains. Lateral ventricles are indicated with an asterisk. Arrow in B indicates axon Probst bundles. (D-F) P0 cortical slices were immunolabeled against GFAP (red) and L1 cell adhesion molecule (green) and counterstained with DAPI (blue). The thickness of the corpus callosum is indicated in D and F with white bars. Arrows in E point at Probst bundles and asterisk indicates lack of corpus callosum. (G-I) P10 slices were immunolabeled against DKK3 (red) and calbindin (CALB; green) and counterstained with DAPI (blue). Note the disorganization of the hippocampus in Emx1-NICD (H). (J-L) Quantifications of cortical thickness (J), corpus callosum thickness (K) and hippocampal area (L). Mean±s.e.m. *P*-values were obtained using an unpaired, two-tailed Student's *t*-test. CA1-CA3, cornu ammonis hippocampal regions; Ctrl, control; DG, dentate gyrus; LV, lateral ventricle. Scale bars: 500 µm (A-C,G-I); 250 µm (D-F).

Analyses of these mice at P0 revealed several telencephalic gross morphological defects. Emx1-NICD brains exhibited enlarged lateral ventricles, aberrant hippocampi, thinner cortices and agenesis of the corpus callosum (Fig. 1A,B,D,E,G,H,J-L). In contrast, Emx1-dnMAML had smaller lateral ventricle volumes, smaller hippocampi and dysgenesis of corpus callosum (Fig. 1C,F,I-L), with stronger deficiencies in the posterior corpus collosum, including misrouted axons (Fig. 1F).

The corpus callosum is a large commissure that connects the right and left hemispheres and is formed by the axons of SATB2^+^ neurons that are found in all cortex layers, but particularly abundant in upper layers (Britanova et al., 2008; Dobreva et al., 2006). Given that Notch signaling regulates neuronal differentiation, one possibility is that the SATB2^+^ neurons are not correctly produced in Emx1-NICD brains, thus resulting in a lack of callosal cells (Alcamo et al., 2008; Srivatsa et al., 2014). To test this hypothesis, we quantified the number of SATB2^+^ neurons in the neocortex of Emx1-NICD mice at P0. Strikingly, Emx1-NICD exhibited increased numbers of SATB2^+^ neurons compared with their littermate controls (2.49-fold increase, *P*=0.004; Fig. S2A,B), indicating that the observed phenotype is not caused by a deficiency in the production of callosal neurons, but possibly due to deficiencies in axon pathfinding or midline defects. In this direction, we observed Probst bundles by Hematoxylin & Eosin (H&E) staining and L1 axon immunolabeling, suggesting that aberrant axon bundles fail to extend across the midline (Fig. 1B,E, arrows). We labeled neurons with mCherry fluorescent protein in E13.5 control and Emx1-NICD embryos by *in utero* electroporation (IUE), when the first SATB2^+^ neurons are born (Ozaki and Wahlsten, 1998), then collected P0 mice for analysis. In control mice, we detected mCherry-labeled axons through the midline. In Emx1-NICD mice, some mCherry^+^ axons extended towards the midline but failed to cross to the other hemisphere, resulting in an aberrant accumulation of axon fascicles (Fig. S2C).

The hippocampus was also affected in both Emx1-NICD and Emx1-dnMAML mice (Fig. 1G-I). The hippocampus is composed of the dentate gyrus (DG), which includes the granule cells (calbindin^+^), and the hippocampus proper, which contains pyramidal neurons (DKK3^+^) (Han et al., 2020; Khalaf-Nazzal and Francis, 2013). Emx1-NICD hippocampi were smaller than the controls and showed a severe disorganization of both cell types at P10, without increased apoptosis at P0 (Fig. 1H,L; Fig. S3). Conversely, upon dnMAML expression, the hippocampus was organized correctly, but all regions were drastically reduced in size (Fig. 1I,L). We also noticed instances of mispositioned DKK3^+^ neurons in the Emx1-dnMAML hippocampi, namely DKK3^+^ cells crossing the upper DG blade and/or ectopically clustering in the hippocampal fissure (Fig. S3D, arrows).

### Notch signaling is not a main mediator of dorsal telencephalic patterning but regulates Cajal–Retzius cell production

**Fig. 2.**
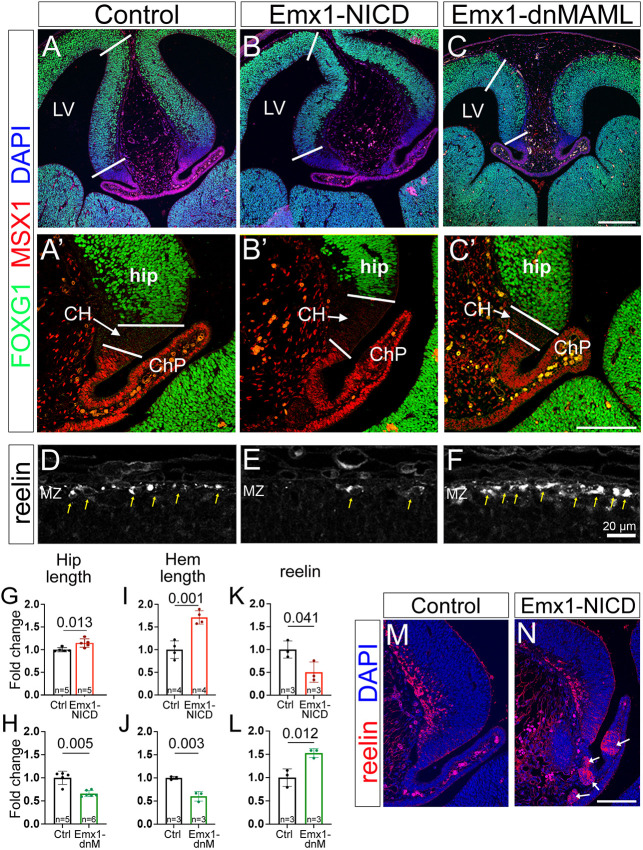
**Midline patterning and production of Cajal–Retzius cells in Emx1-NICD and Emx1-dnMAML telencephalons.** (A-C′) E13.5 sections were immunolabeled against MSX1 (red) and FOXG1 (green), and counterstained with DAPI (blue). White bars indicate hippocampal primordia (A-C) and cortical hem (A′-C′) regions. (D-F) E13.5 cortical sections were immunolabeled for reelin (white) to identify Cajal-Retzius cells (yellow arrows). (G-L) Quantifications of hippocampal length (G,H), cortical hem length (I,J) and number of reelin^+^ cells/area (K,L) shown as fold change compared with each control littermate. Mean±s.e.m. *P*-values were obtained using an unpaired, two-tailed Student's *t*-test. (M,N) Emx1-NICD mice show patches of ectopic reelin^+^ cells at E13.5 (arrows). CH, cortical hem; ChP, choroid plexus; Ctrl, control; hip, hippocampal primordia; LV, lateral ventricle; MZ, marginal zone. Scale bars: 250 µm (A-C); 100 µm (A′-C′,M,N); 20 µm (D-F).

During the course of central nervous system patterning, the dorsal telencephalic midline becomes organized into three distinct regions: the choroid plexus (ChP), the cortical hem (CH) and the hippocampal primordium, which is contiguous with the neocortex (Subramanian and Tole, 2009) (Fig. 2A,A′).

Because both Emx1-NICD and Emx1-dnMAML mice exhibit abnormal hippocampi and enlarged or smaller lateral ventricles, respectively, we hypothesized that dorsal telencephalic midline patterning could be affected in our models. Consistent with this idea, a triple knockout of the Notch signaling effectors *Hes1*, *Hes3* and *Hes5* exhibits defects in ChP development (Imayoshi et al., 2008). To identify possible patterning alterations, we labeled E13.5 coronal sections with FOXG1 and MSX1 to distinguish the hippocampal primordium, CH and ChP regions (Fig. 2A-C′). Emx1-NICD mice exhibited elongated hippocampi and CH, whereas these structures were significantly shorter in Emx1-dnMAML, compared with their littermate controls (Fig. 2A-C′,G-J). These changes could be reflecting alterations in cell cycle dynamics, but the general patterning and localization of these territories is maintained.

An important function of the CH is the production of Cajal–Retzius cells, which secrete the extracellular glycoprotein reelin (Soriano and Del Rio, 2005), which is essential for cortical and hippocampal neuron migration and lamination (Borrell et al., 2007; Gonzalez-Billault et al., 2005; Reyes et al., 2022; Sekine et al., 2011; Simo and Cooper, 2013; Simo et al., 2010). Because the size of the CH is altered in both Emx1-NICD and Emx1-dnMAML mice, we labeled and quantified the number of Cajal–Retzius cells, using reelin as a marker. Although the CH is larger in Emx1-NICD brains, we found a reduction in reelin^+^ cells in both the cortical marginal zone (Fig. 2D,E,K; Fig. S4A,C) and the hippocampus (Fig. S4B). Interestingly, despite fewer reelin^+^ cells present in the cortex and hippocampus, we identified ectopic patches of reelin^+^ cells within the ChP of Emx1-NICD mice (Fig. 2M,N). In order to confirm whether these cells were indeed Cajal–Retzius cells or cells that had aberrantly upregulated the expression of reelin, we tested two other Cajal–Retzius markers: calretinin (calbindin 2) and TBR1 (Hevner et al., 2003). Notably, the ectopic reelin^+^ cells expressed calretinin but not TBR1 (Fig. S4F-H‴). Emx1-dnMAML mice showed a significant increase in reelin^+^ cells at E13.5 in the cortical marginal zone (Fig. 2C,L), although no changes were observed in the hippocampus (Fig. S4D).

### Early-born projection neuron production is limited by Notch signaling during neocortical development

**Fig. 3.**
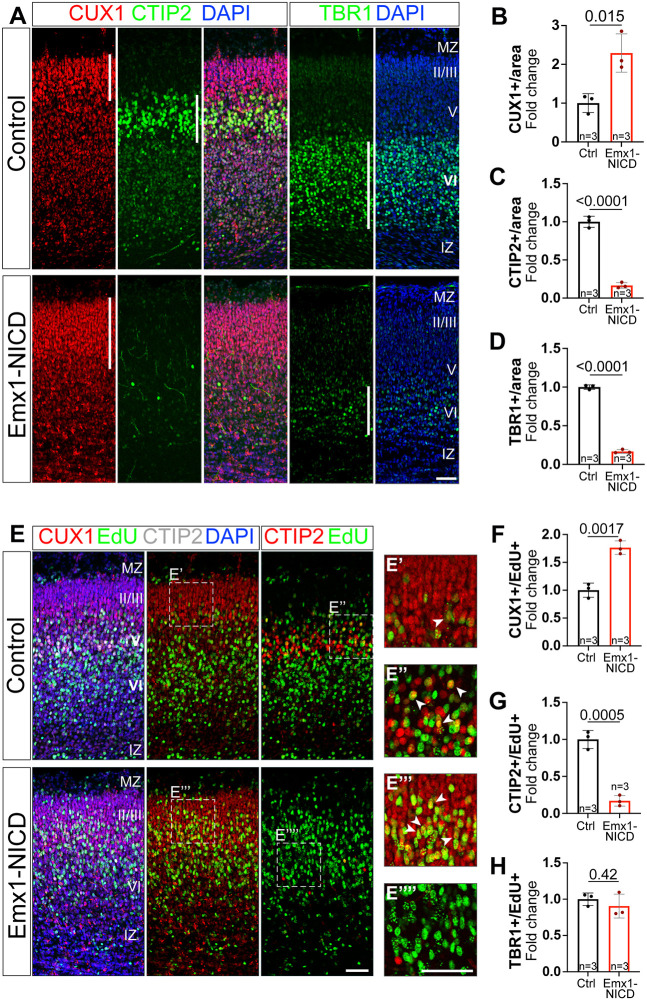
**Emx1-NICD neocortices exhibit increased ratios of upper-layer neurons.** (A) P0 cortical brain section immunolabeled with CUX1 (red), CTIP2 (green) and TBR1 (green) antibodies, and counterstained with DAPI (blue). (B-D) Quantifications of the number of cells per area are shown as fold change compared with control littermates. (E-E⁗) P0 cortical section labeling of EdU (green), CUX1 (red), CTIP2 (white left, red right), counterstained with DAPI (blue). Arrowheads indicate EdU^+^/CUX1^+^ cells (E′,E‴) or EdU^+^/CTIP2^+^ cells (E″). Note the absence of EdU^+^/CTIP2^+^ cells in E⁗. (F-H) Quantifications of the number of cells per area shown normalized to their corresponding control littermates. Mean±s.e.m (B-D,F-H). *P*-values were obtained using an unpaired, two-tailed Student's *t*-test. IZ, intermediate zone; MZ, marginal zone. Scale bars: 50 µm.

Because our Emx1-NICD animals showed increased numbers of SATB2^+^ cells, we assessed whether other cortical cell types were also affected by NICD overexpression. To avoid staining or counting biases, we used a semi-automatic cell counter platform (RapID; Sekar et al., 2021) and we normalized each quantification to their corresponding littermate controls. In Emx1-NICD cortices, we observed a dramatic reduction of CTIP2 (BCL11B)^+^ and TBR1^+^ neurons and an increase in CUX1^+^ cells in comparison with controls (Fig. 3A-D). Despite the significant changes in the ratio of cell populations in these cortices and the reduction in reelin^+^ cells, lamination was largely normal, with CUX1^+^ projection neurons located at the top of the cortex and TBR1^+^ neurons located in the most apical layer of the cortical plate (Fig. 3A).

To investigate further the changes in cell populations, we performed birth-dating experiments using 5-ethynyl-2′-deoxyuridine (EdU) to label dividing progenitors. We injected EdU at E13.5 and then analyzed the cortices at P0 to assess the fate outcomes of the EdU-labeled progenitors. The number of neurons that were both EdU^+^ and CUX1^+^, CTIP2^+^ or TBR1^+^ were quantified and normalized to the total number of EdU^+^ neurons (Fig. 3E-H). Whereas in control brains EdU-labeled E13.5 neural progenitor cells mostly gave rise to CTIP2^+^ layer V neurons (Fig. 3E-E⁗,G), we observed a strong decrease of EdU^+^ CTIP2^+^ cells in Emx1-NICD brains. Concomitantly, the presence of EdU^+^ CUX1^+^ cells increased almost twofold in Emx1-NICD brain in comparison with their control littermates (Fig. 3E-F). At this stage of development, RGs have already passed beyond the period of production of layer VI neurons, and we did not observe a significant change in EdU^+^ TBR1^+^ cell production (Fig. 3H).

**Fig. 4.**
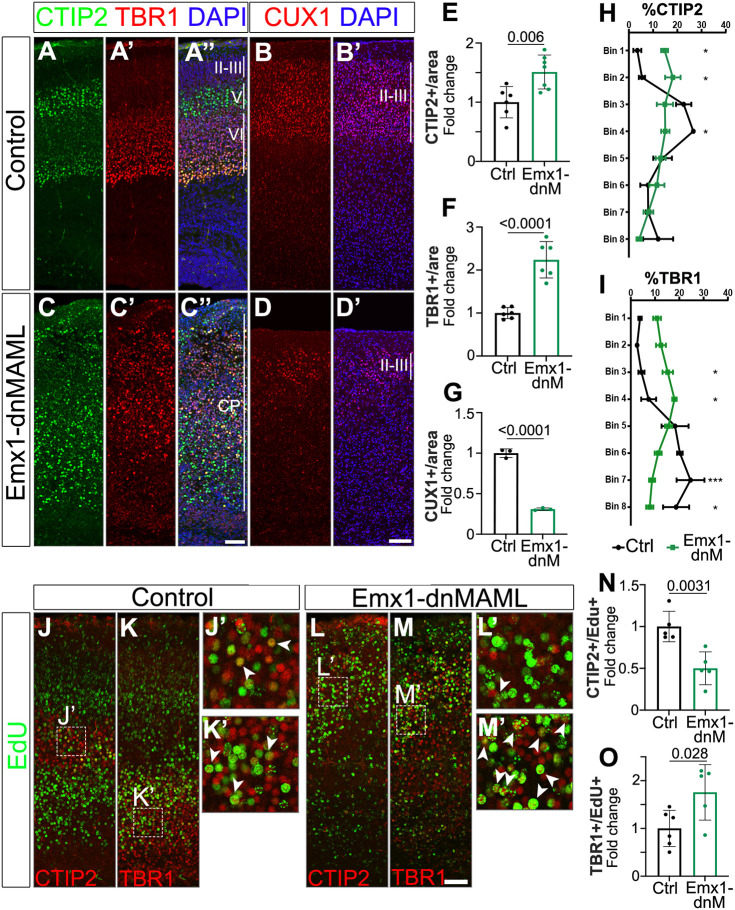
**Emx1-dnMAML neocortices exhibit increased numbers of deep-layer neurons and lamination defects.** (A-D′) P0 cortical coronal section immunolabeled with CTIP2 (green) and TBR1 (red) (A-A″,C-C″) and P10 coronal section immunolabeled with CUX1 (red) (B,B′,D,D′) antibodies. Tissues were counterstained with DAPI (blue). (E-G) Quantifications of the number of cells per area are shown as fold change compared with control littermates. (H,I) Distribution of CTIP2^+^ (H) and TBR1^+^ (I) cells in control (Ctrl) and Emx1-dnMAML (dnM) in P0 cortical brain slices. (J-M′) P0 cortical section labelings of EdU (green), CTIP2 (red) and TBR1 (red), counterstained with DAPI (blue). Arrowheads indicate EdU^+^/CTIP2^+^ cells (J′,L′) or EdU^+^/TBR1^+^ cells (K′,M′). (N,O) Quantifications of the number of cells per area normalized to their corresponding control littermates. Mean±s.e.m. *P*-values in E-G,N,O were obtained using an unpaired, two-tailed Student's *t*-test. For the cell distributions in H,I, multiple unpaired *t*-tests (one per bin) with Welch correction were performed (*P<0.05; ***P<0.001; adjusted *P*-values). CP, cortical plate. Scale bars: 50 µm.

Next, we evaluated the consequences of blocking Notch signaling in the developing cortex using the Emx1-dnMAML model. Unfortunately, the anti-CUX1 antibody that we were using (Santa Cruz Biotechnology) was discontinued and we had to switch to a new vendor (Proteintech). The new antibody only labels CUX1^+^ neurons efficiently after P10; thus, we switched the age of all subsequent CUX1 analyses from P0 to P10. In this case, we found a significant decrease in CUX1^+^ projection neurons, whereas CTIP2^+^ and TBR1^+^ neurons were over-represented (Fig. 4A-G). We also performed birth-dating experiments to measure whether the changes in cortical neuron composition are linked to changes in the timing of neurogenesis, similar to the experiments described before. We found a significant decrease in EdU^+^ CUX1^+^ neurons and corresponding increases in EdU^+^ CTIP2^+^ and EdU^+^ TBR1^+^ neurons, suggesting that the neural progenitors in the Emx1-dnMAML mice continue to produce TBR1^+^ neurons beyond the normal time window of layer VI neurogenesis (Fig. 4J-O).

In contrast with the normal lamination observed in Notch GOF, Emx1-dnMAML mice neocortices also showed severe disruption of the cortical layers in the dorsomedial region with milder lamination defects in the lateral aspects of the neocortex (Fig. S5). To quantify this phenotype, we divided the cortical plate into eight bins and counted the number of CTIP2^+^ and TBR1^+^ neurons in each bin. In control animals, TBR1^+^ neurons were mainly positioned at the bottom of the cortical plate, as expected (bins 6-8 contained 63.5% of all TBR1^+^ neurons), and CTIP2^+^ cells were enriched in more basal locations (bins 3-4 contained 49.2% of all CTIP2^+^ cells). Conversely, both CTIP2^+^ and TBR1^+^ neurons were dispersed across the whole thickness of the cortical plate in Emx1-dnMAML samples (bins 6-8 contained only 27.8% of all TBR1^+^ cells and bins 3-4 included 29.9% of CTIP2^+^ neurons) (Fig. 4H,I).

Despite MAML having clear roles in the Notch signaling pathway, several studies suggest broader functions for MAML1 as a co-factor for multiple signaling pathways, including Wnt, Hippo and sonic hedgehog pathways (Alves-Guerra et al., 2007; Kim et al., 2020; Quaranta et al., 2017; Zema et al., 2020). To determine whether the defects observed in Emx1-dnMAML mice are caused by the role of MAML in the MAML–RBPJ–NICD trimeric protein complex, we generated a Notch1 conditional knockout line by crossing Notch1^f/f^ mice (Yang et al., 2004) with the Emx1-CRE driver (hereafter Notch1cKO). Similar to Emx1-dnMAML1 mice, Notch1cKO mice exhibited dysgenesis of the corpus callosum and smaller lateral ventricles and hippocampi (Fig. S6A-D). We also observed thinner cortices that had considerably reduced upper layers (II-III) and dispersed cortical neurons, but this phenotype was not as severe as in the Emx1-dnMAML model (Fig. S6E,F). We performed birth-dating experiments as described above. Experiments showed a 2.4-fold increase in TBR1^+^ population production (i.e. TBR1^+^ EdU^+^) compared with littermate controls, whereas no differences were observed for the CTIP2^+^ population (Fig. S6G-J). These data indicate that the Notch1cKO phenotype closely mimics that of Emx1-dnMAML, suggesting that the overall defects we observed in Emx1-dnMAML mice are mainly due to the imbalance downstream of Notch.

Given the migration defects observed in both loss-of-function models, we hypothesized that defects in the RGs could be contributing to these phenotypes, as described previously in Emx1-RBPJ^f/f^ mice (Son et al., 2020). We labeled RGs using nestin at E13 and P0 (Fig. S7). Surprisingly, we did not observe changes in the organization or distribution of the RGs. At P0, we observed a depletion of nestin signal in the most medial region of the cortex but not in the lateral aspects in the Emx1-dnMAML model, suggesting a possible depletion of ventricular progenitors at this age.

### Notch signaling regulates radial glia cell cycle dynamics

**Fig. 5.**
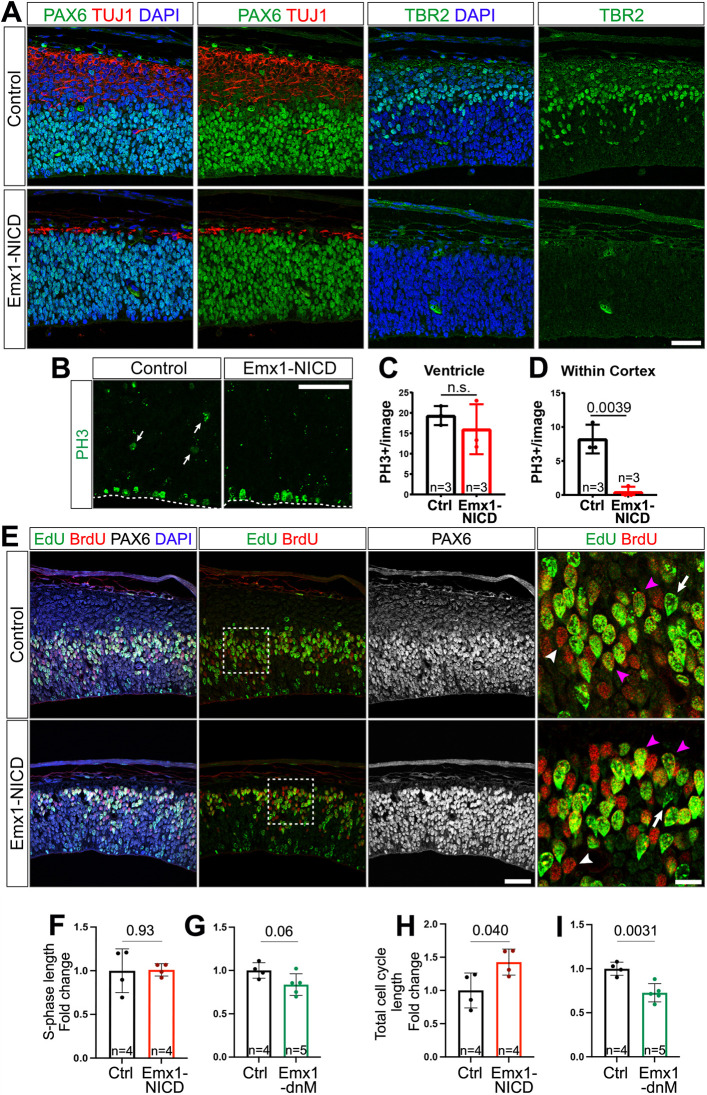
**Notch regulates radial glia cell cycle dynamics.** (A) Cortical slices from E13.5 control and Emx1-NICD embryos immunostained against PAX6 (green), TUJ1 (red) and TBR2 (green) and counterstained with DAPI (blue). (B) Cortical slices from E13.5 control and Emx1-NICD embryos immunostained against phospho-Histone3 (PH3, green). Arrows indicate PH3^+^ cells away from the ventricular surface, likely being intermediate progenitors. Dashed line delineates the ventricular surface. (C,D) Quantification of PH3^+^ cells in the ventricular surface (C) and anywhere else in the cortex area above the ventricular surface (D). (E) E13.5 cortical section labelings of EdU (green), BrdU (red) and PAX6 (white), counterstained with DAPI (blue). White arrows indicate EdU^+^/BrdU^−^ cells; white arrowheads indicate EdU^−^/BrdU^+^ cells; magenta arrowheads indicate EdU^+^/BrdU^+^ cells. Boxed areas are shown at higher magnification in the far-right panels. (F,G) Quantification of S-phase length in Emx1-NICD (F) and Emx1-dnMAML (dnM) (G) mice in comparison with their control littermates. (H,I) Quantification of total cell cycle length in Emx1-NICD (H) and Emx1-dnMAML (dnM) (I) mice in comparison with their control littermates. Mean±s.e.m (C,D,F-I). *P*-values were obtained using an unpaired, two-tailed Student's *t*-test. Ctrl, control; n.s., not significant. Scale bars: 50 µm (A,B,E); 20 µm (right-hand panels in E).

Notch signaling is required for maintaining the progenitor pool, and HES genes downstream of Notch repress bHLH transcription factors, especially those with proneural functions (Dennis et al., 2019; Huang et al., 2014; Imayoshi et al., 2010; Jadhav et al., 2006; Kageyama et al., 2009; Maurer et al., 2014; Nieto et al., 2001). Overexpression of Delta1, HES1 or activated Notch1 prolongs mitotic activity in different types of progenitor cells (Austin et al., 1995; Del Bene et al., 2008; Dorsky et al., 1995; Kageyama et al., 2009). For these reasons, we characterized the cortical neural progenitors in our transgenic models at E13.5. We observed decreased numbers of TUJ1 (TUBB3)^+^ post-mitotic neurons at E13.5 in Emx1-NICD mice, as expected (Fig. 5A, left), but we also found that all Emx1-NICD embryos exhibit a complete depletion of TBR2^+^ (EOMES) intermediate progenitors (Fig. 5A, right). To confirm that the intermediate progenitors were absent, as opposed to a downregulation of TBR2 protein, we labeled all mitotic cells with phosphohistone H3 (PH3). Although we did not observe any changes in the number of mitotic cells adjacent to the ventricle (RGs), the basally located PH3^+^ cells (intermediate progenitors) were absent in Emx1-NICD mice (Fig. 5B-D). In order to determine whether the lack of TBR2^+^ progenitors observed is a developmental delay or a permanent loss of intermediate progenitors, we extended these analyses to E15.5. Interestingly, at E15.5, we observed TBR2^+^ cells at ratios similar to those observed in the controls (Fig. S8), suggesting that the lack of intermediate progenitors at E13.5 reveals a developmental delay.

To characterize further the cell cycle dynamics in our different genetic models, we measured the length of the cell cycle using dual-window labeling with the thymidine analogs EdU and 5-bromo-2'-deoxy-uridine (BrdU), as described previously (Harris et al., 2018). Pregnant mice at E13.5 were injected with a pulse of EdU followed by a pulse of BrdU 2 h later. All animals were euthanized 30 min after the second pulse (150 min total) and the tissues were processed and stained for EdU, BrdU and PAX6. Because NICD mice were missing the TBR2^+^ intermediate progenitors, we limited the quantification to PAX6^+^ apical RGs. RGs labeled by EdU but not BrdU (PAX6^+^ EdU^+^ BrdU^−^) exited S phase during the 2-h period between pulses. The ratio of PAX6^+^ EdU^+^ BrdU^−^ cells over the total number of cells in S phase (PAX6^+^ EdU^+^) equals 2 h/time of S phase (2 h/Ts). The ratio between the number of cells in S phase at any given time point (PAX6^+^ BrdU^+^) and the total PAX6^+^ proliferating population is proportional to the ratio Ts/total cell cycle time (Ts/Tc). Using these parameters, we estimated the percentage of cells in S phase for each sample and then calculated the average Ts and Tc, normalizing each value to their littermate controls to avoid staining or imaging biases.

Although the length of S phase was not significantly altered in any of the models (9.02 h in control, 9.12 h in Emx1-NICD and 9.20 h in Emx1-dnMAML; Fig. 5E-G), the total length of the cell cycle was significantly longer in Emx1-NICD progenitors (7.9 h longer or 42±3.8% increase, *P*=0.040) and shorter in Emx1-dnMAML RGs (6.7 h shorter or 38±4.7% increase, *P*=0.003).

These data together show that activation of Notch signaling results in depletion of intermediate progenitors at early time points, but not at E15.5, and lengthening of the cell cycle without affecting the total length of S phase.

### Overactivation of Notch signaling results in transcriptomic changes of Notch effectors, bHLH transcription factors and the miRNAs let-7, miR-99a/100 and miR-125b

**Fig. 6.**
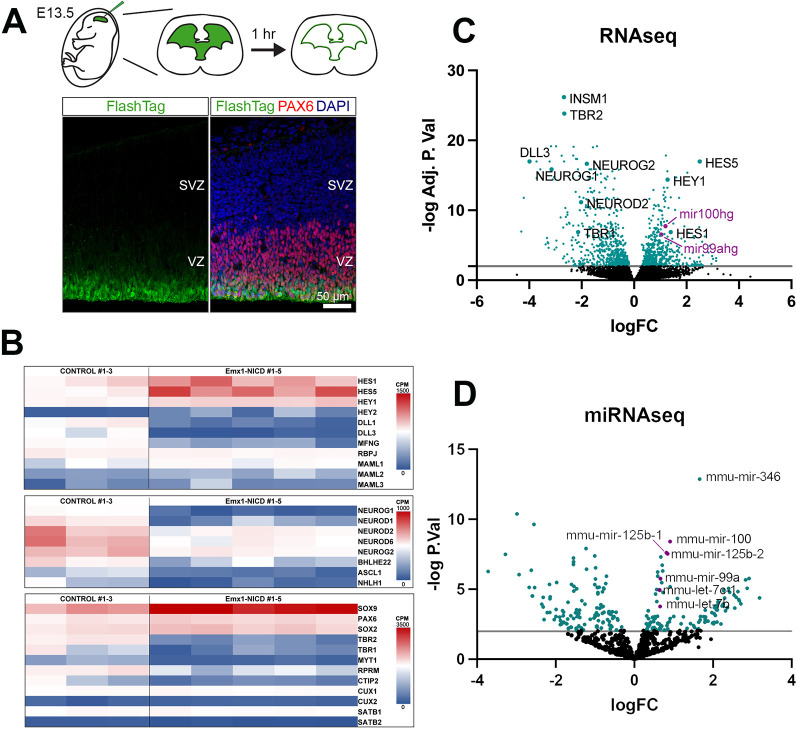
**Transcriptional changes in Emx1-NICD radial glia.** (A) Top: Experimental design: a CFSE-containing solution (green) was injected into the 3rd ventricle of E13.5 embryonic mice (left). CFSE diffused through the ventricular system (middle), labeling all cells surrounding the ventricles (green line, right). Bottom: PAX6^+^ RGs (red) closest to the ventricular surface were labeled with FlashTag (green) 1 h post-injection. (B) Heatmaps showing RNAseq analysis of selected Notch signaling-related genes (top), bHLH genes (middle) and cortical markers (bottom). (C,D) Volcano plots representing differential gene (C) and miRNA (D) expression between Emx1-NICD (*n*=5) and control (*n*=3) RGs samples. Ratio of counts per million between Emx1-NICD and control per gene or miRNA is plotted. The *x*-axis represents the logarithmic fold ratio of Emx1-NICD/control per gene and miRNA identified. The *y*-axis represents the logarithmic adjusted *P*-value (false discovery rate) calculated by the Benjamini–Hochberg Procedure. Genes with fewer than five counts per million reads in all samples were filtered prior to analysis, leaving 12,556 genes. miRNAs present in fewer than three samples were filtered prior to analysis, leaving 779 miRNAs.

To identify the downstream targets of Notch activation that may play a role in neurogenesis and/or cell cycle regulation within the progenitor population, we profiled E13.5 RG transcriptomes using RNA sequencing (RNAseq), facilitated by specific labeling of RGs using FlashTag (Govindan et al., 2018). As described previously, FlashTag utilizes carboxyfluorescein esters (CFSEs) that, when injected into the ventricles, label cells in contact with the cerebrospinal fluid. Because the apical RGs are transiently in contact with the ventricle walls during mitosis, this technique allows for specific labeling of the RG population. We confirmed that 1 h after injection all FlashTag-labeled cells were PAX6^+^ RGs (Fig. 6A). Next, we used FlashTag to label RGs in control (*n*=3) and Emx1-NICD (*n*=5) littermate embryos at E13.5. We dissected the neocortices 1 h post-injection, isolated the labeled RGs using fluorescence-activated cell sorting (FACS), and performed RNAseq (Han et al., 2023). Multidimensional scaling analysis showed a clear clustering of all the Emx1-NICD samples (Fig. S9A). Gene ontology (GO) enrichment analyses using PANTHER revealed that processes over-represented in Emx1-NICD cortices include the terms: ‘regulation of Notch signaling pathway’ (GO:0008593, *P*=0.00135), ‘negative regulation of cell differentiation’ (GO:0045596, *P*=2.22×10^−9^), ‘cell fate commitment’ (GO:0045165, *P*=1.73×10^−8^) and ‘regulation of cell cycle’ (GO:0051762, *P*=0.00026); processes downregulated include the terms: ‘neuron differentiation’ (GO:0030182, *P*=4.36×10^−17^), ‘forebrain development’ (GO:0030900, *P*=1.65×10^−10^) and ‘axon guidance’ (GO:0007411, *P*=0.00148) (Fig. S9B).

As expected, known Notch effectors, such as *Hes1*, *Hes5*, *Hey1* and *Hey2*, were upregulated by NICD, whereas the Notch receptors *Dll1* and *Dll3*, were downregulated along with *Mfng* (manic fringe homolog), a glycosyltransferase that modulates Notch activity (Fig. 6B,C). HES and HEY genes negatively regulate the expression of proneural bHLH transcription factors in several contexts and, accordingly, we observed a reduction in *Neurog1*, *Neurog2*, *Neurod1*, *Neurod2*, *Neurod6* and *Ascl1* (Fig. 6B,C). Although we only analyzed the RG transcriptome, we observed a significant downregulation of some deep-layer markers (e.g. *Tbr1*, *Myt1* and *Rprm*), layer V genes (*Ctip2*) and intermediate progenitor markers (e.g. *Tbr2*), but we did not observe differences in upper-layer markers (e.g. *Cux1* and *Satb2*).

Strikingly, we also observed upregulation of *miR100hg* and *miR99ahg*, which are the host genes for two miRNA clusters (Fig. 6C, purple data points). *miR100hg* (miR-100 host gene) includes miR-100, let-7a-2 and miR-125b-1, and *miR99ahg* (mir-99a host gene) encodes for miR-99a, miR-125b-2 and let-7c. Upregulation of these miRNAs in Emx1-NICD samples was further confirmed by miRNA sequencing of FlashTag-labeled purified RGs (Fig. 6D; Table S2), as described before. To confirm the sequencing results, we performed single-molecule *in situ* hybridization (RNAscope) of miR99ahg; as expected, miR99ahg expression was increased in Emx1-NICD samples compared with controls (Fig. S10).

### Let-7, miR-125b and miR-99a/100 are required downstream of Notch to restrict early-born projection neuron fates

**Fig. 7.**
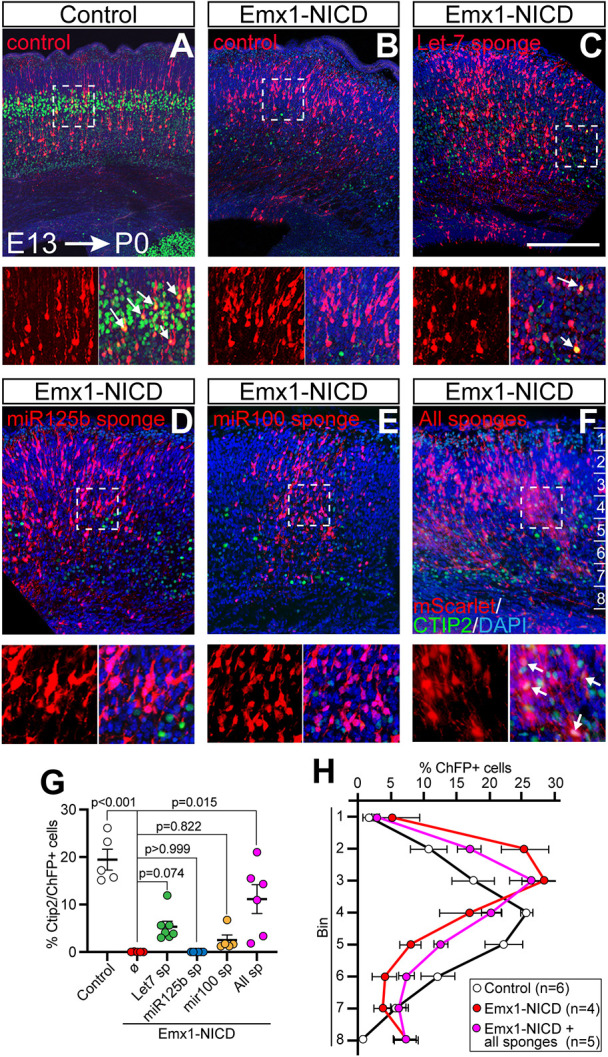
**Inhibition of let-7, miR-125b and miR-100 expression rescues cortical cell fate defects in Emx1-NICD mice.** (A-F) Representative images of control (A) and Emx1-NICD (B) mice electroporated with control plasmid (mScarlett, red) and Emx1-NICD mice co-electroporated with mScarlett and miRNA sponges against let-7 (C) miR-125b (D), miR-100 (E), or all three sponges (F). Electroporations were performed on E13.5 embryos and electroporated brains were collected at P0 (E13→P0). DAPI was used as counterstain. Boxed areas are shown at higher magnification below. Arrows indicate mScarlet^+^/CTIP2^+^ cells. (G) Quantification of the percentage of CTIP2^+^/mScarlet^+^ cells in each condition. Mean±s.e.m. Adjusted *P*-values were obtained with Kruskal–Wallis test and Dunn's post-hoc test. (H) Distribution of mScarlet^+^ cells from the electroporations shown in A, B and F. Mean±s.e.m. Adjusted *P*-values were obtained with Kruskal–Wallis test and Dunn's post-hoc test. Scale bar: 250 µm.

To test whether the upregulation of let-7, miR-125b and/or miR-99a/100 play any roles in the cortical phenotypes observed, we performed IUE using microRNA sponges (Barta et al., 2016; La Torre et al., 2013) to inhibit miRNA activity in Emx1-NICD mice, and we analyzed the consequent effects on cell fate. Plasmids expressing specific miRNA sponges together with an mScarlet plasmid were electroporated into Emx1-NICD E13.5 embryos, and the electroporated brains were collected at P0 (Fig. 7A-F). These samples were processed, labeled with CTIP2 antibodies, and the numbers of CTIP2^+^ mScarlet^+^ cells were determined. Whereas in control animals we observed 19.4%±8.4 of CTIP2^+^ mScarlet^+^ /mScarlet^+^ cells (*n*=5), we did not observe any CTIP2^+^ mScarlet^+^ cells in Emx1-NICD brains (*n*=4) (Fig. 7A,B), in agreement with our data showing that upon NICD overexpression E13.5 RGs generate upper-layer cells instead of layer V neurons. We did not observe any significant changes when let-7, miR-125b or miR-100 were inhibited [5.56%±2.9 (*n*=7), 0% (*n*=5) and 2.83%±2.6 (*n*=6), respectively; Fig. 7D,E,G]. Notably, when we electroporated the three sponges together (let-7, miR-125b and miR-100) in Emx1-NICD mice, significantly more electroporated cells were CTIP2^+^ (11.15%±7.4; Fig. 7F,G). Similarly, we quantified the distribution of the electroporated cells throughout the cortical plate (Fig. 7H). In control samples, most of the electroporated cells were located in bins 4-5 whereas NICD overexpression shifted the cells to bins 2-3. Inhibition with the three sponges reduced the number of cells in the upper bins (*P*=0.041). Together, these data suggest that these miRNAs are downstream effectors of Notch signaling and necessary to produce upper-layer neurons, possibly through epistatic mechanisms.

## DISCUSSION

### Multifaceted functions of Notch during telencephalic morphogenesis

The Notch pathway has long been recognized for its roles in cell specification, patterning, differentiation and regeneration (Artavanis-Tsakonas et al., 1999; Bosze et al., 2020; Conner et al., 2014; Imayoshi et al., 2010; Jadhav et al., 2006; Maurer et al., 2014; Nelson et al., 2007; Riesenberg et al., 2009; Sestan et al., 1999). However, its contributions to telencephalic development remain unresolved. Utilizing GOF and DN transgenic mouse lines, we demonstrate that balanced Notch signaling is required for hippocampal and corpus callosum development, and we also show that Notch is a key regulator of neurogenesis in the neocortex.

Given the vast array of gross morphological defects observed in both GOF and DN models, we examined whether Notch regulates the patterning of the dorsal telencephalic midline, perhaps affecting the development of the hippocampus and ChP, changing brain fluid homeostasis and the volume of the ventricles. Although there are obvious differences in the size of the hippocampal, CH and ChP areas in the transgenic mice, the establishment of the different territories is not affected by Notch changes.

Although the ChP is properly patterned and establishes a sharp boundary with the CH, we identified ectopic reelin^+^ cells in the ChP region at E13.5 in Emx1-NICD brains. Notably, these ectopic cells express reelin and calretinin (both markers of Cajal–Retzius cells), but not TBR1, a marker normally detected in Cajal–Retzius cells at E13.5, but not expressed in these cells at later stages (Hevner et al., 2003). A previous study using lineage-tracing analysis of the prospective ChP region indicated that these progenitors sequentially give rise to reelin^+^ Cajal–Retzius cells first and later to non-neural ChP epithelial fates (Imayoshi et al., 2008). Inactivation of *Hes1*, *Hes3* and *Hes5* genes led to an enhanced development of Cajal–Retzius cells at the expense of ChP cell fates, suggesting that Notch signaling regulates the progenitor transition from cells that produce Cajal–Retzius cells to progenitors that will generate non-neural ChP epithelial cell identities. However, around E12.5, the levels of HES1 and HES5 are downregulated after ChP cell fate specification (Imayoshi et al., 2008). The presence of ectopic reelin^+^ cells suggests that this later downregulation of Notch signaling may be required to maintain the ChP fate and that ChP progenitors can transdifferentiate to neural fates, or at least express some neural markers, upon sustained HES1/5 activity. The ectopic reelin^+^ cells observed could alternatively be the consequence of aberrant migratory patterns from the CH. In fact, Notch has been shown to regulate migration patterns in the cortex through interactions with the reelin-Dab1 signaling pathway (Hashimoto-Torii et al., 2008).

Alternatively, Notch could also be affecting the tangential expansion of the progenitor pool, indirectly affecting the volume of the ventricles. In some large mammals, including humans, the cerebral cortex undergoes a massive expansion that takes place by a growth in surface area rather than in thickness and is the basis for gyrencephaly (Del-Valle-Anton and Borrell, 2022). In this direction, recent work has shown that local disruption of Notch signaling can trigger cortical folding (Han et al., 2021).

### Balanced Notch signaling is essential for the development of the corpus callosum

Transgenic models overexpressing *Hes5* in the neocortex (Bansod et al., 2017) and *Hes1/Hes3/Hes5* and *Rbpj* knockouts has been described previously (Imayoshi et al., 2010, 2008; Son et al., 2020), but, to our knowledge, our study is the first to report corpus callosum defects upon alterations of Notch signaling in mice.

In Emx1-NICD mice, we observed an increase of SATB2^+^ cells together with the presence of Probst bundles, indicating that callosal axons are present but unable to cross the midline. One possibility is that the defects in the corpus callosum may be directly caused by the changes in the cortical cell ratios observed in our different models. The pioneering axons, the first axons to cross the midline during development, are known to guide later axons and experimental approaches indicate that later axons are unable to find the correct path in the absence of pioneering axons (Koester and O'Leary, 1994). SATB2^+^ neurons are normally detected from E13.5 as CUX1^+^ upper layer neurons, but a small percentage colocalize with CTIP2 or TBR1 (Alcamo et al., 2008). Thus, it is possible that the lack of CTIP2^+^ SATB2^+^ or TBR1^+^ SATB2^+^ pioneering axons is causing this phenotype. Alternatively, deficiencies in the midline glia (glial wedge and indusium griseum), which secrete guidance cues, or changes in the expression of the appropriate receptors in the SATB2^+^ neurons could also result in the failure to cross the midline. In this direction, our RNAseq results indicate that several guidance receptors are altered upon NICD overexpression, including *Slit1*, *Dcc1* (*Dscc1*), *Unc5a*, *Plxna4* and *Nrp1* (Table S1), even though our analyses were restricted to RGs and not postmitotic neurons.

### Notch regulates radial glia cell cycle length and cortical neurogenesis

In the present study, we show that the switch between generating deep to upper-layer projection neurons is regulated by Notch, as RGs labeled with EdU generated upper-layer fates sooner in Emx1-NICD mice and they generated deep-layer cells for longer periods upon the expression of dnMAML compared with their respective littermate controls. These results parallel phenotypes observed in *Hes5* knockouts and *Hes5*-overexpression models, in which the timing of neurogenesis was also affected (Bansod et al., 2017).

Because the length of the cell cycle is longer in Emx1-NICD RGs, the fate acquisition changes do not seem to correlate with the number of cell divisions. However, the length of the cell cycle and the timing of cell cycle exit could be influencing these neurogenic fate decisions. For example, during bristle patterning in *Drosophila*, Notch signaling controls cell cycle progression, such that cells with elevated Notch signaling divide first whereas those with lower signaling extend their G2 phase, making them more sensitive to lateral inhibition and consequently change their cell fate (Hunter et al., 2016). Our data could fit a similar model in which NICD extends RG cell cycle time, making these cells more susceptible to fate determinant factor(s).

Although effects driven by the ectopic expression of NICD or dnMAML in post-mitotic neurons cannot be completely ruled out, our data support the idea that fate decisions are decided at the progenitor stage. In this direction, pro-neural bHLH transcription factors, a family of transcriptional regulators known to play key roles in fate determination, are expressed in the terminal cell cycle of neural progenitors from S phase or G2 (Prasov and Glaser, 2012), and are known to regulate both cell cycle exit and fate choices (Castro et al., 2011; Maurer et al., 2018; Nguyen et al., 2006). Surprisingly, we also detected the presence of transcripts normally associated with specific subpopulations of postmitotic neurons in our sequencing experiments using purified RGs. As suggested before, low levels of these mRNAs in RGs may not lead to detectable protein expression but might prime the cells for differentiation upon cell cycle exit. Although we distinguished significant differences in the expression of intermediate progenitor markers (*Tbr2*) and deep-layer genes (*Tbr1*, *Myt1*, *Ctip2*), we did not observe any differences in upper-layer markers (*Cux1*, *Satb2*). Published reports have shown that TBR1/CTIP2 and TBR1/FEZF2 initially overlap in their expression before genetic repression and depression networks establish the distinct layer subtype identities (Toma et al., 2014). For instance, FEZF2 is a transcriptional repressor that acts by repressing genes that would be inappropriate for layer V, including *Tbr1* (Srinivasan et al., 2012) and layer II-IV genes (Tsyporin et al., 2021). Our data suggest that the reduction in deep-layer genes in RGs may be affecting the expression of upper-layer genes later (i.e. upon cell cycle exit).

### miRNAs downstream of Notch are required for upper-layer neuron fate acquisition

We identified two miRNA clusters – *miR100hg* and *miR99ahg* – with increased expression in Emx1-NICD cortices. Both clusters encode for let-7, miR-125b and miR-99a/100. let-7 and miR-125b are essential regulators of developmental timing in various organisms (Ambros and Horvitz, 1984; Pasquinelli et al., 2000; Wu et al., 2012). In the mammalian retina, let-7 and miR-125b regulate the switch from progenitors that produce early cell fates to retinal progenitors that yield late-born cell fates (La Torre et al., 2013; Xia and Ahmad, 2016). In the cortex, let-7 has been recognized as an important factor for maintenance of homeostasis (Fernandez et al., 2020) and is vital in the generation of late cell types (Patterson et al., 2014; Shu et al., 2019). Moreover, we have recently shown that let-7 also regulates progenitor cell cycle dynamics in the cortex (Fairchild et al., 2019). Although there is limited literature on the roles of miR-99a/100 in the central nervous system, the miR-99a/100, let-7, miR-125b tricistrons have been shown to regulate hematopoietic progenitor homeostasis (Emmrich et al., 2014).

Strikingly, the inhibition of the activity of these miRNAs using specific miRNA sponges is sufficient to partially rescue the Emx1-NICD phenotype. We have previously shown that let-7 inhibition leads to a shortening of the S/G2 phase of cell cycle (Fairchild et al., 2019), but further inquiry is needed to discern whether the effects of these sponges are mediated by the regulation of cell cycle or through other downstream targets. A widely recognized target of let-7 is the chromatin remodeler HMGA2 (Lee and Dutta, 2007), an important regulator of neocortical neurogenesis (Shu et al., 2019). let-7 also regulates the levels of the nuclear receptor TLX (NR2E1) (Zhao et al., 2010) and the cell cycle genes cyclin D1, cyclin D2, CDK4, CDK6 and CDC25A (Bueno and Malumbres, 2011). Further experiments aimed at understanding the molecular mechanisms downstream of let-7 and the possible cooperative activities between the different miRNAs will shed light on the machinery that instructs cortical fate acquisition.

Importantly, *miR100hg* is located in a human chromosome region (11q24.1) deletion of which is associated with Jacobsen syndrome (JBS, OMIM #147791). This syndrome involves intellectual disability, abnormal head shape, microphthalmia, and increased likelihood of autism spectrum disorders (Akshoomoff et al., 2015; Favier et al., 2015; Guerin et al., 2012). The deletions associated with JBS range from 7 to 20 Mb. The heterogeneity of phenotypes supports the hypothesis that JBS is a contiguous gene deletion syndrome in which the loss of different combinations of genes causes particular phenotypes. Owing to the rarity of reported cases, it has not yet been possible to tease out the requirements of individual genes, including *miR100hg*. Notably, a rare microtriplication of 1.8 Mb in the 11q24.1 region (partial trisomy), which includes *miR100hg* and only a handful of other genes (11 in total), also results in intellectual disability with severe verbal impairment (Beneteau et al., 2011). Thus, a further understanding of the roles that *miR99ahg* and *miR100hg* play in telencephalic development will also allow us to gain further insights into the molecular underpinnings of developmental brain disorders.

## MATERIALS AND METHODS

### Animals

All animals were used with approval from the University of California Davis Institutional Animal Care and Use Committees and housed and cared for in accordance with the guidelines provided by the National Institutes of Health. B6N.129-*Gt(ROSA)26Sor^tm1(MAML1)Wsp^*/J (ROSA26^loxP-stop-loxP-dnMAML1^) and *Notch1^tm2Rko^*/GridJ (Notch1^f/f^) were generous gifts from Dr Ivan Maillard and Dr Raphael Kopan, respectively. *Gt(ROSA)26Sor^tm1(Notch1)Dam^*/J (ROSA26^loxP-stop-loxP-Notch1-ICD^) and B6.129S2-*Emx1^tm1(cre)Krj^*/J (Emx1-Cre) mice were obtained from The Jackson Laboratory. All animals are currently available at The Jackson Laboratory [#008159 (Murtaugh et al., 2003), #032613 (Tu et al., 2005), #006951 (Yang et al., 2004) and #005628 (Gorski et al., 2002), respectively]. To drive NICD and dnMAML expression in the developing mouse telencephalon, ROSA26^loxP-stop-loxP-Notch1-ICD^ or ROSA26^loxP-stop-loxP-dnMAML1^ were crossed with Emx1-Cre/+ mice. To generate Emx1-Cre/+; Notch1^f/f^ (Notch1cKO) mice, Notch1^f/f^ were crossed with Emx1-Cre*/+* mice to generate an intermediate stock and then Emx1-Cre/+; Notch1^f/+^ were bred with Notch1^f/f^.

### Constructs

The MSCV puro let-7 sponge was a gift from Dr Phil Sharp (Addgene plasmid #29766) (Kumar et al., 2008), the pRNA-U6-let-7 sponge was a gift from Dr Phillip Zamore (Addgene plasmid #35664), and the MG-miR-125b-sponge-bulge was a gift from Dr David Baltimore (Addgene plasmid #45790) (Chaudhuri et al., 2012). The miR-100 sponge was designed using the miRNAsong algorithm (Barta et al., 2016) (5′- CACAAGTTCGGATCTACGGGTTAATTCACAAGTTCGGATCTACGGGTT-3′). miR-100 sponge sequences were cloned in tandem into a pCAG backbone (Simo and Cooper, 2013) to obtain a 12mer miR-100 sponge sequences. miR-100 sponge is expected to also target miR-99a based on sequence homology.

### Histology and immunohistochemistry

Brains were collected at the indicated ages and prepared for cryoembedding or paraffin embedding. Samples to be cryoembedded were fixed in 3.7% formalin/PBS by submersion overnight at 4°C, cryoprotected with 30% sucrose/PBS solution, embedded in Optimum Cutting Temperature (OCT) compound (Tissue-Tek), and quickly frozen using dry-ice. Samples for paraffin embedding were fixed in modified Carnoy's fixative (ethanol, formaldehyde and acetic acid) overnight at 4°C, dehydrated, cleared with xylene, and embedded in paraffin blocks. OCT embedded brain blocks were cryo-sectioned (15 µm) and paraffin embedded blocks were sectioned (5 µm), both on a coronal plane. Immunostainings were performed on free-floating cryoprotected sections and mounted paraffin sections with agitation. Paraffin sections were deparaffinized using xylene, rehydrated with ethanol, and rinsed with 0.3% Triton X-100/PBS. Antigen retrieval was performed on all samples with hot (95°C) 0.01 M sodium citrate pH 8 for 20 min. Some samples required further antigen retrieval, which included an additional acid wash (2 N HCl and 0.5% Triton X-100/PBS) for 1 h at room temperature. All sections were then blocked with 0.1% Triton X-100/PBS and either 5% milk or 10% normal donkey serum for 1 h at room temperature. Blocking solution was used for primary antibody incubation (overnight, 4°C). After primary antibody incubation, free-floating sections were washed three times (10 min each) in 0.1% Triton X-100/PBS and mounted sections were washed five times (5 min each) in PBS. Species-specific, fluorescently labeled secondary antibodies were used in blocking solution (60-90 min, room temperature). 4′,6-diamidino-2- phenylindole (DAPI) (Sigma-Aldrich) was used for nuclear staining. See Table S3 for details of antibodies and working dilutions. Images were taken using a Fluoview FV3000 confocal microscope (Olympus) or Axio Imager.M2 with Apotome.2 microscope system (Zeiss). All images were assembled using Photoshop and Illustrator (Adobe).

### *In utero* electroporation

*In utero* microinjection and electroporation was performed at E13.5 as described previously (Simo and Cooper, 2013), using timed pregnant Emx1-NICD mice. For control electroporations, DNA solutions containing 0.5 mg/ml pCAG-ChFP plasmids were mixed in 10 mM Tris, pH 8.0, with 0.01% Fast Green, and 1 µl of the solution was injected per embryo. Tweezertrodes electrodes (BTX) with 5-mm pads were used for electroporation (five 50 ms pulses of 30 V). To express the miRNA sponges, a solution containing 1 mg/ml of each sponge individually, or combined, and 0.5 mg/ml pCAG-ChFP was used. All experimental manipulations were performed in accordance with protocols approved by the University of California, Davis Institutional Animal Care and Use Committee (IACUC). At P0, electroporated brains were collected and processed as described.

### FlashTag NSC labeling and FACS

Labeling of cortical neuronal progenitors with CFSEs was achieved as described elsewhere (Govindan et al., 2018). Briefly, 1 µl of a 5 mM solution of CellTrace CFSE (from the CellTrace CFSE Cell Proliferation Kit, Invitrogen, C34554) and 0.01% Fast Green in DMSO was injected into the third ventricle of E13.5 control and Emx1-NICD embryos. Dams were allowed to recover and injected embryos were collected 1 h post-injection. Embryonic cortices were dissected individually and dissociated into single cells using Papain Dissociation System (Worthington Biochemical, LK003150) following the manufacturer's protocol. Cells were resuspended in FACS media (DMEM/F12 without phenol red supplemented with 10% fetal bovine serum and B-27) and sorted using a Beckman Coulter Astrios EQ Cell Sorter.

### RNA and miRNA sequencing

Total RNA from control (*n*=3) and Emx1-NICD (*n*=5) sorted cells was extracted using the Total RNA Purification Plus Kit (Norgen Biotek Corp., 48300). Gene expression profiling was carried out using a 3'-Tag-RNA-Seq protocol. Barcoded sequencing libraries were prepared using the QuantSeq FWD kit (Lexogen) for multiplexed sequencing according to the recommendations of the manufacturer using both the UDI-adapter and UMI Second-Strand Synthesis modules (Lexogen). The fragment size distribution of the libraries was verified via micro-capillary gel electrophoresis on a LabChip GX system (PerkinElmer). Barcoded miRNA-Seq libraries were prepared using the NEXTflex Small RNA Sequencing kit V3 (PerkinElmer) with sequence randomized adapters according to the recommendations of the manufacturer. The fragment size distribution of the libraries was verified via micro-capillary gel electrophoresis on a Bioanalyzer 2100 (Agilent). Both sets of libraries were quantified by fluorometry on a Qubit instrument (Life Technologies), and then pooled in equimolar ratios. The library pools were quantified by qPCR with a Kapa Library Quant kit (Kapa Biosystems/Roche). Finally, both library sets were sequenced on a HiSeq 4000 sequencer (Illumina) with single-end 100 bp reads.

### EdU and BrdU labeling

For EdU birth-dating experiments, pregnant dams were injected intraperitoneally with 25 mg EdU/kg body weight at E13.5 and pups were sacrificed at birth. For the EdU/BrdU dual window labeling experiments, pregnant dams were injected intraperitoneally with 12.5 mg/kg body weight of EdU at E13.5 and injected again with 12.5 mg/kg body weight of BrdU after 2 h. Brains were collected 30 min post BrdU injection. EdU was detected following manufacturer instructions (Thermo Fisher Scientific, C10337). Prior to detection of BrdU by immunofluorescence as previously described, tissue was treated with hot (95°C) 0.01 M sodium citrate pH 8 for 20 min followed by an acid wash (2 N HCl and 0.5% Triton X-100/PBS) for 1 h at room temperature.

### Statistical methods

Specific number of biological replicates and statistical methods used are specified in each figure or figure legend. For cortical thickness, the thickness of the somatosensory cortex in three consecutive brain slices was measured and averaged per brain. The same strategy was used to measure the corpus callosum thickness. The hippocampal area was measured from both hippocampal hemispheres of three sections containing the dorsal hippocampus in which the habenula was visible. These measures were averaged and represented the results for a single brain. For histological and IUE quantifications, single or double fluorescently labeled cells were quantified for at least three consecutive sections in each brain and their results averaged.

To measure cell distribution in the cortex we used RapID (Sekar et al., 2021). Briefly, a grid containing eight equally sized bins was manually placed in the cortex with bin 1 mainly covering the marginal zone and bin 8 covering the intermediate zone. The quantification of fluorescently labeled cells was automatically determined by the software.

All statistical analyses and plot generation were performed using Prism 9 (GraphPad).

## Supplementary Material

10.1242/develop.201408_sup1 Supplementary information
