# Supplementary material for: Notch directs telencephalic development and controls neocortical neuron fate determination by regulating microRNA levels
Source: Development. 2023 Jun 5;150(11):dev201408. doi: 10.1242/dev.201408 (PMC10309580; doi:10.1242/dev.201408)
Supplement: Supplementary information [file develop-150-201408-s1.pdf]

Supplementary Figure 1

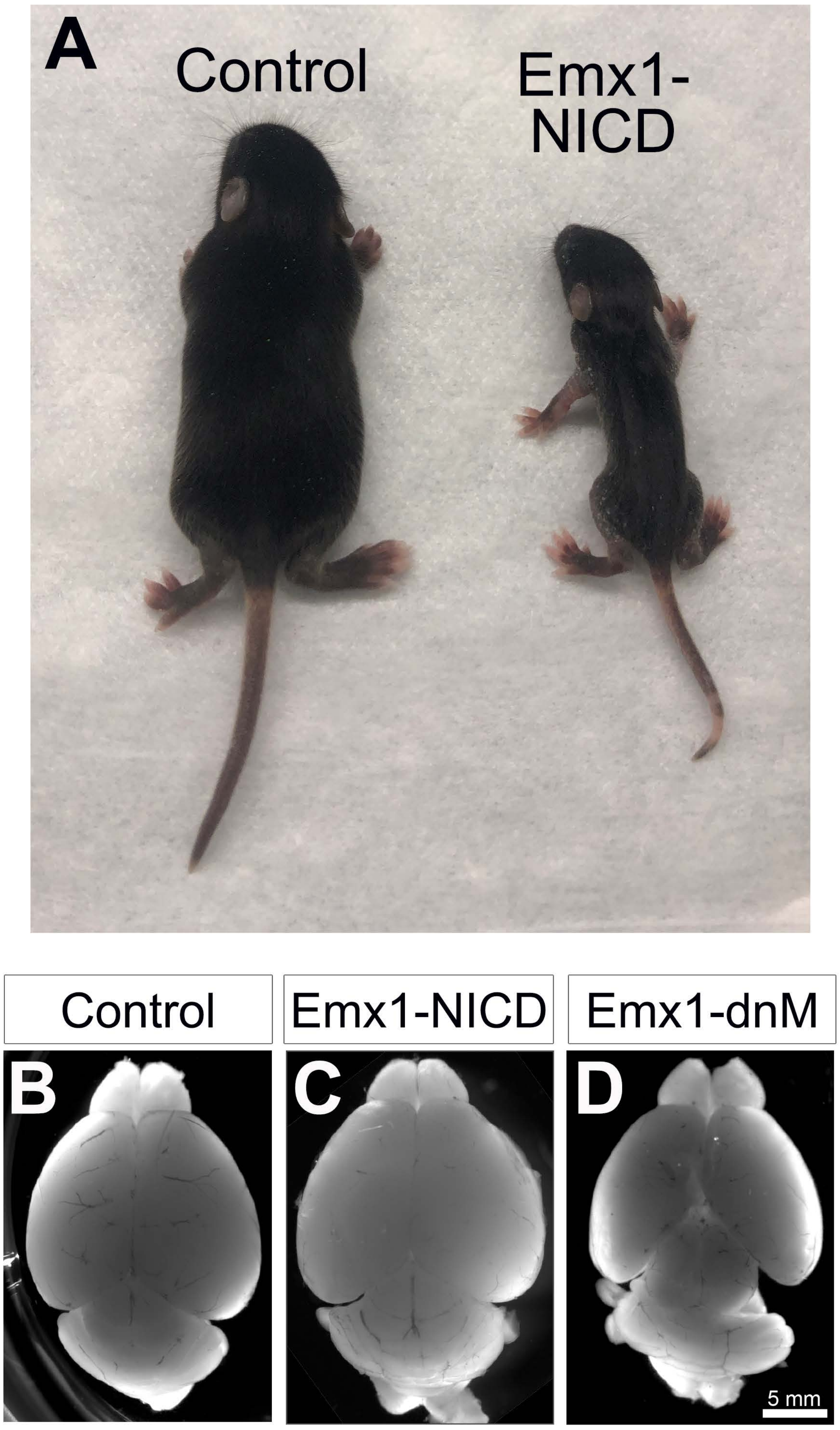

Fig. S1. Emx1-NICD and Emx1-dnMAML mice

A. At P14, Emx1-NICD mice are smaller in size compared to their control littermates. **B.** At P0, the size of the brain is significantly smaller in Emx1-dnMAML mice compared to their littermate controls but no significant differences were observed in Emx1-NICD. Scale bar B-D: 5mm.

Supplementary Figure 2

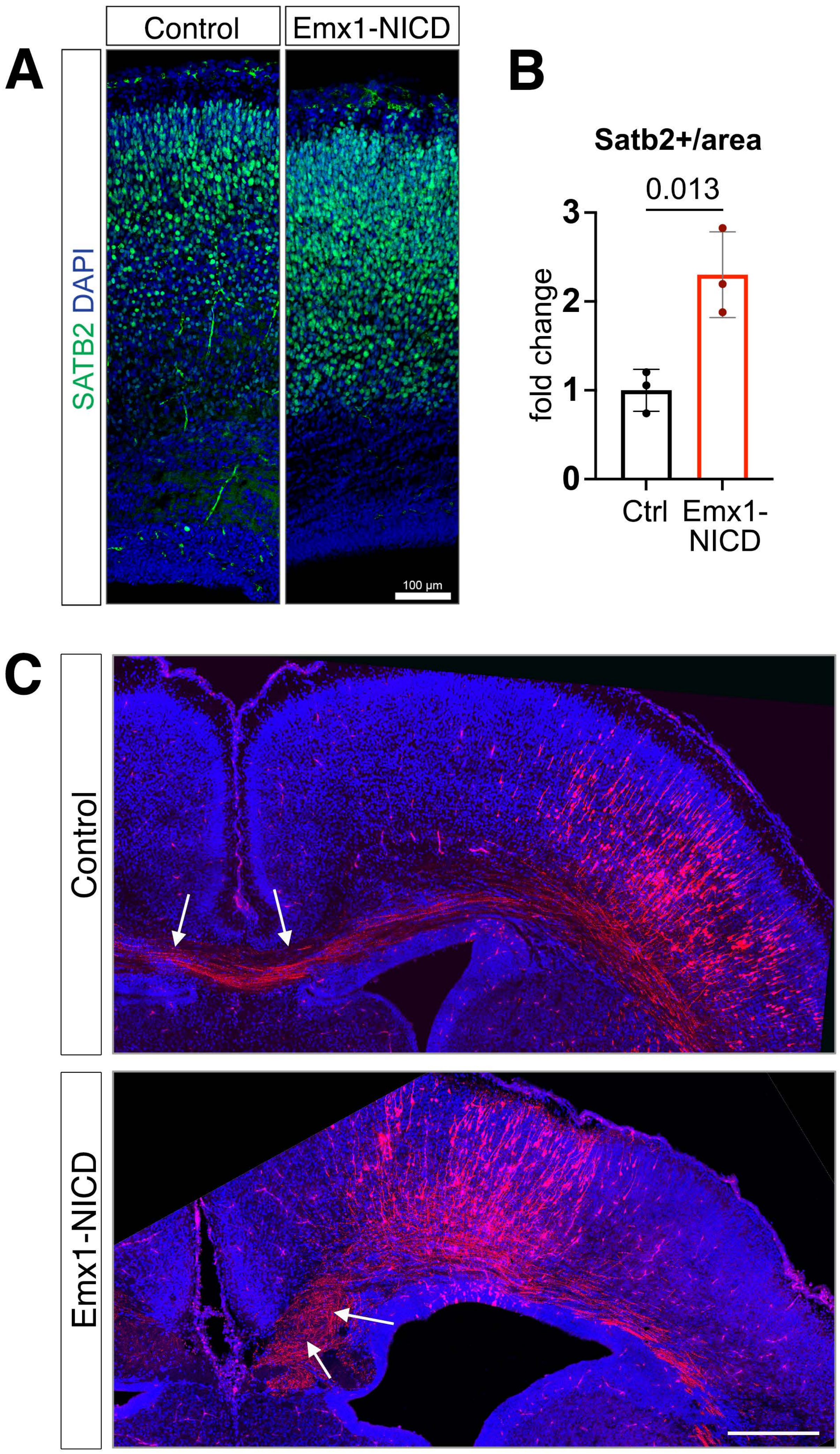

Fig. S2. Agenesis of the corpus Emx1-NICD mice

A-B. At P0, Emx1-NICD cortices exhibit increased ratios of SATB2+ callosal neurons (green) compared to their littermates, DAPI (blue) was used for counterstaining. P-value was obtained using Student's T-test. Scale bar: 100 microns. **C.** Upon *in utero* electroporation of mCherry (red), labeled axons (while arrows) cross the midline in control animals but result in aberrant bundles in Emx1-NICD mice. Scale bars: 100µm A-B; 250µm C.

Development • Supplementary information

Supplementary Figure 3

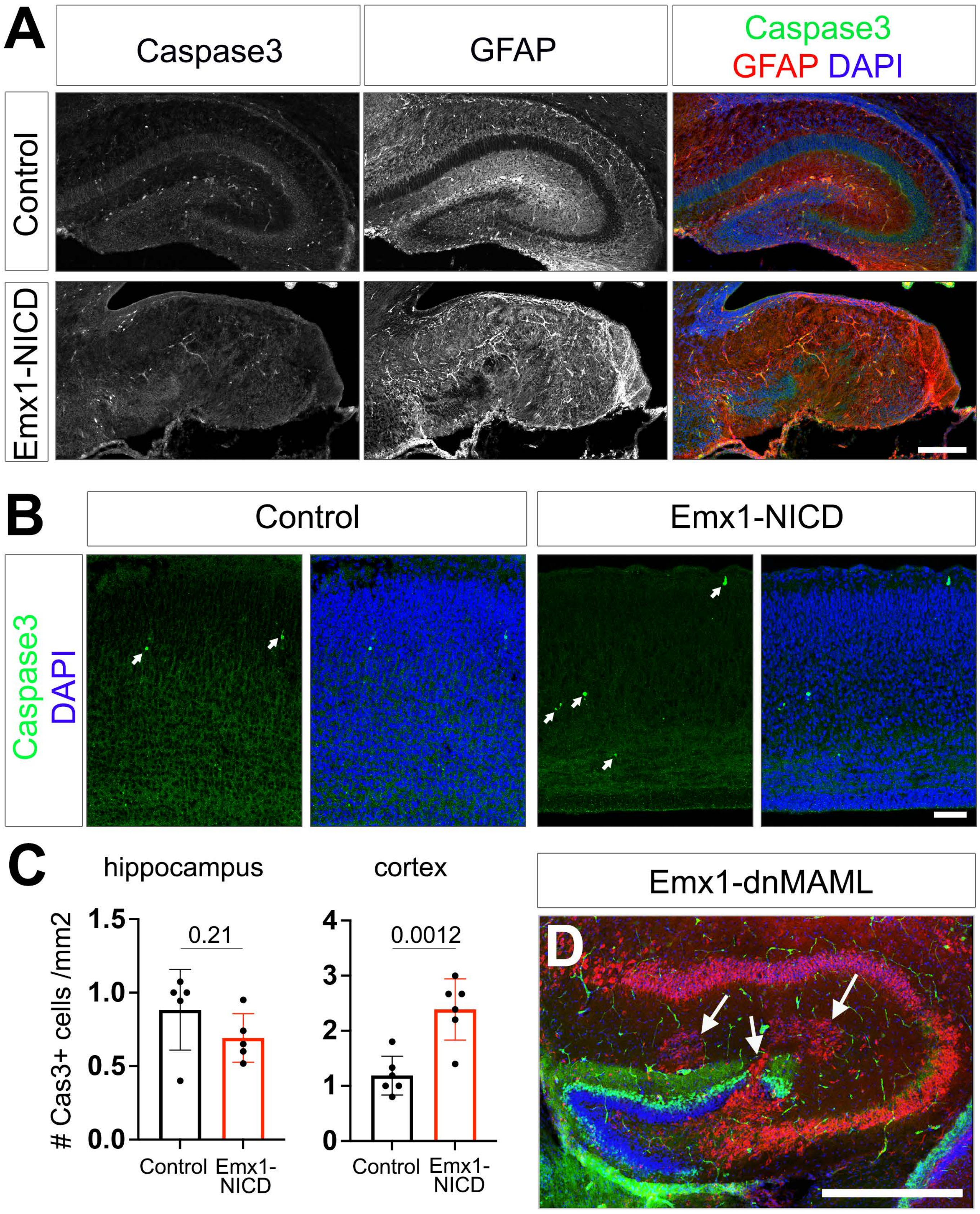

Fig. S3. Hippocampal defects in Emx1-NICD and Emx1-dnMAML mice

A. Caspase3 (green) and GFAP (red) immunolabeling of control and Emx1-NICD hippocampi at P0, counterstained with DAPI (blue). Scale bar: 100 microns. **B.** Caspase3 (green) immunolabeling of control and Emx1-NICD neocortices at P0. White arrows point at Caspase3+ cells. Scale bar: 70 microns. **C.** Quantification of Caspase3+ cells in hippocampus and cortex, p-values were obtained using Student's T-test. **D.** Immunolabeling using DKK3 (red) and Calbindin (green) antibodies, counter-stained with DAPI (blue) of Emx1-dnMAML hippocampal section. Note the ectopic location of some DKK3+ cells (white arrows). Scale bars: 100μm A, 70μm B, 200μm D.

Development • Supplementary information

Supplementary Figure 4

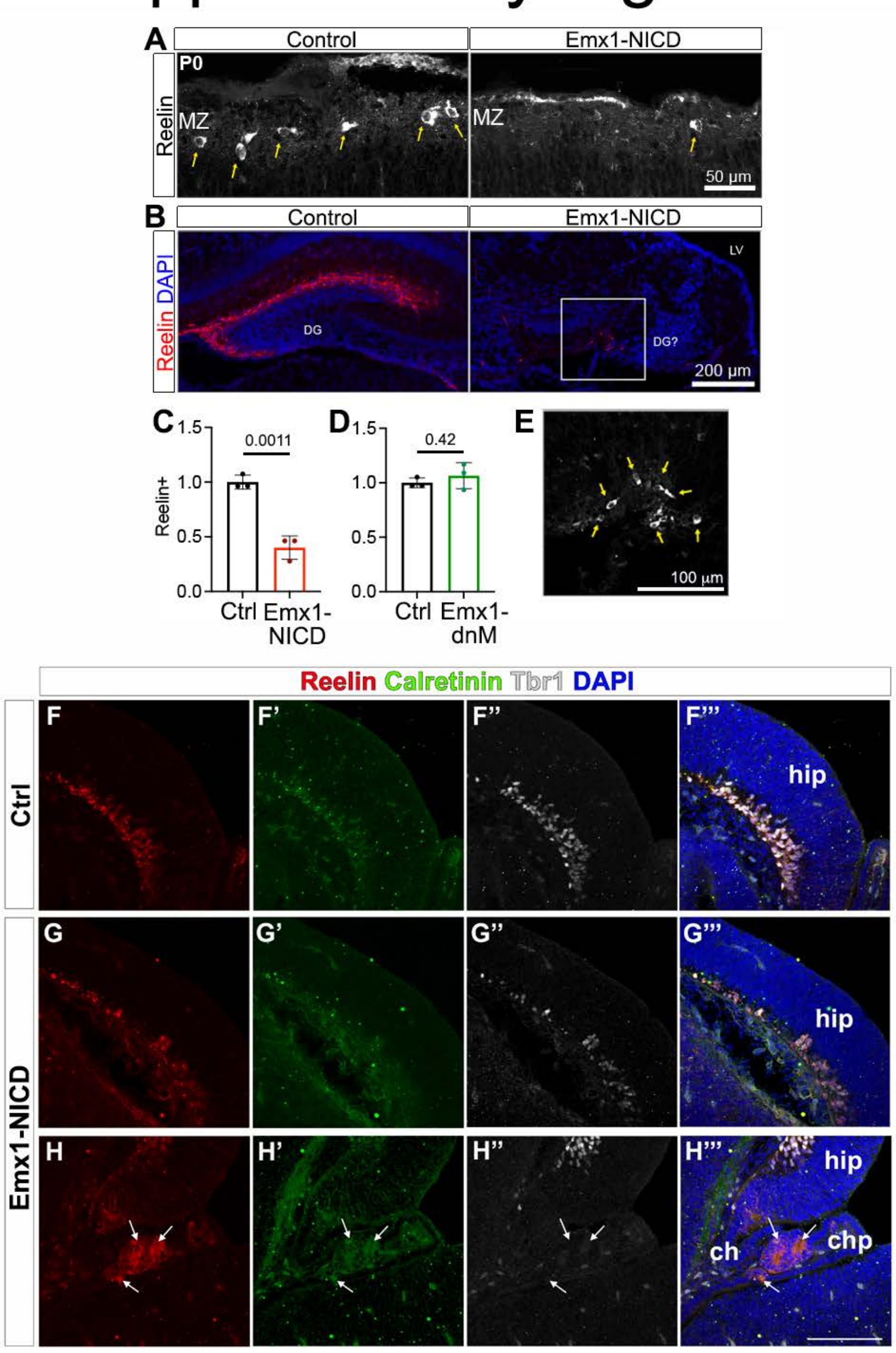

Fig. S4. Aberrant production of Cajal-Retzius cells in Emx1-NICD and Emx1- dnMAML mice A. Emx1-NICD cortices exhibit less Reelin+ (white label, yellow arrows) than their littermate controls at P0. Scale bar: 50 microns. MZ: marginal zone. **B**. Immunolabeling with Reelin (red) in control and Emx1-NICD hippocampal sections. The white inset box is shown in E. LV: lateral ventricle, DG: dentate gyrus. Scale bar: 200 microns. **C-D.** Quantification of number of Reelin+ cells/area in Emx1-NICD and Emx1-dnMAML hippocampi at P0. P-Values were obtained using Student's T-tests. **E.** Only a handful of Reelin+ cells (white, noted with yellow arrows) are detected in Emx1-NICD hippocampi. **......** . Emx1-NICD hippocampi have fewer Cajal-Retzius cells compared to controls at E13.5. Cajal-Retzius cells are detected using a combination of Reelin, Calretinin, and Tbr1 markers. Ectopic patches are detected in the choroid plexus regions of Emx1-NICD mice, which co-express Calretinin but not TBR1. Hip: hippocampal primordia; CH: cortical hem; ChP: choroid plexus. Scale bars: 50µm A, 200µm B, 100µm **E** and **F-H'''**.

Development • Supplementary information

Supplementary Figure 5

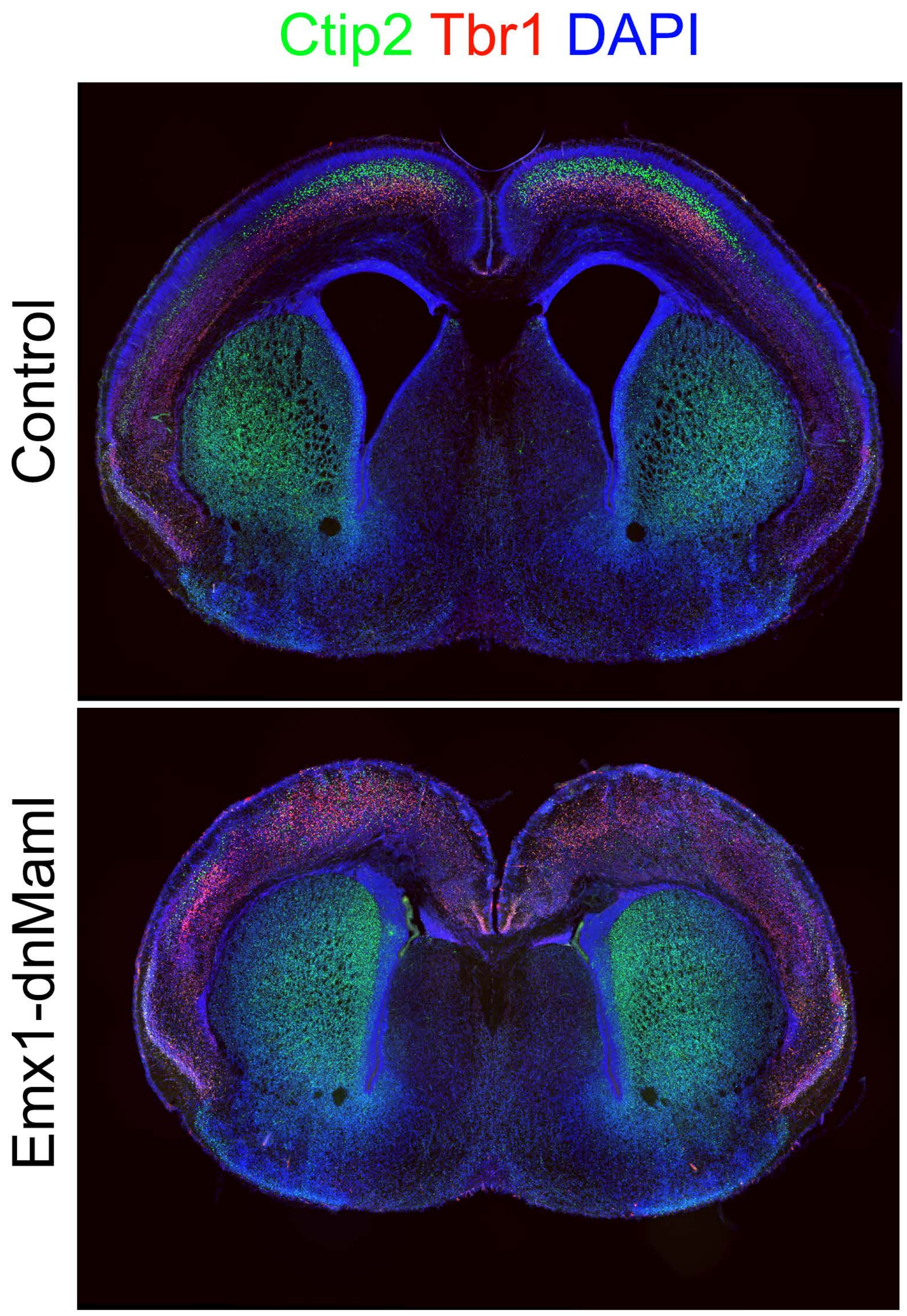

Fig. S5. Lamination defects in Emx1-dnMAML neocortices

Brain coronal sections immunolabeled against CTIP2 (green), TBR1 (red), and counterstained with DAPI (blue). Scale bar: 500μm.

Development • Supplementary information

Supplementary Figure 6

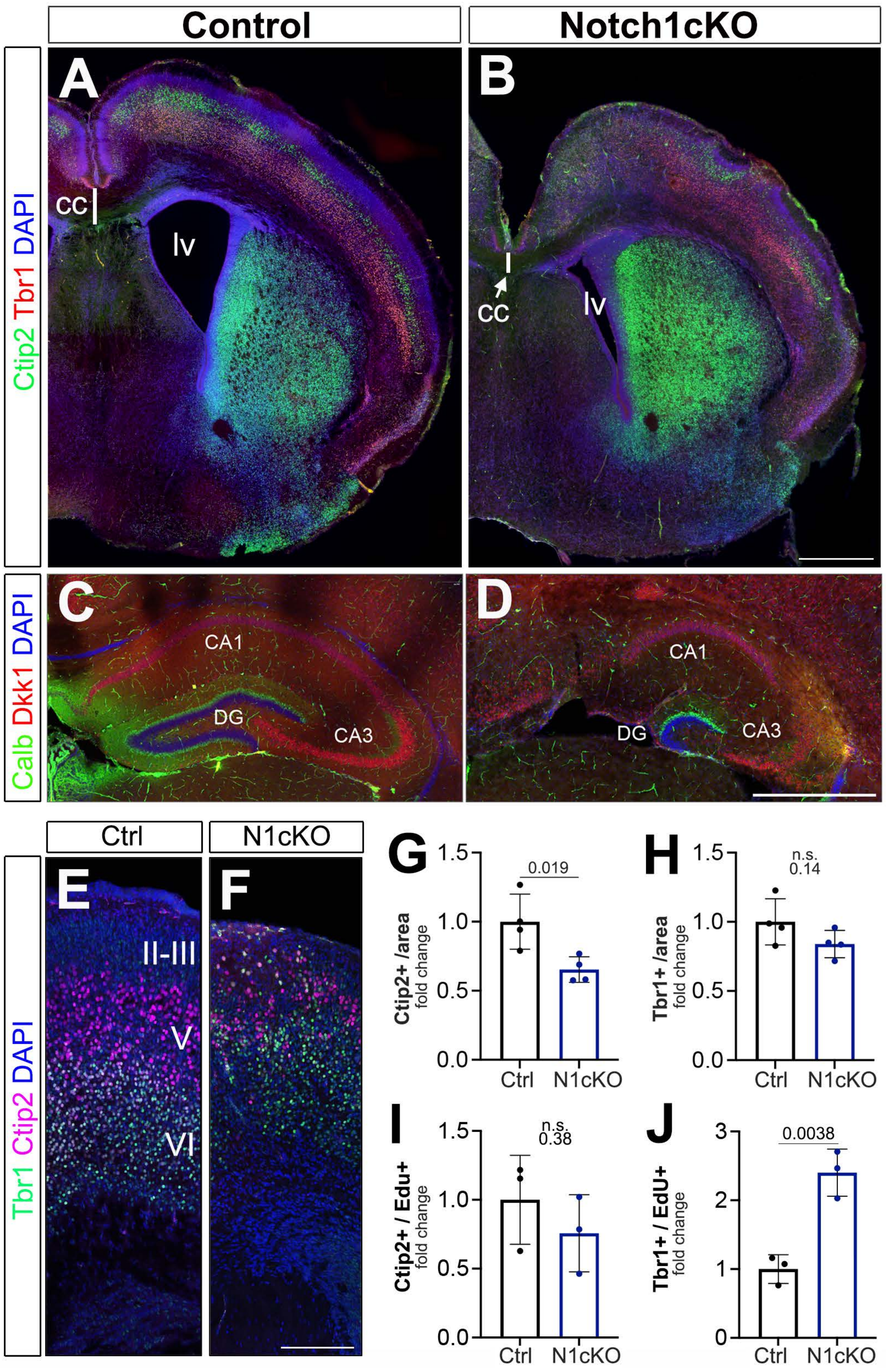

Fig. S6. Notch1cKO phenotypes

A-B. Brain coronal sections immunolabeled against CTIP2 (green), TBR1 (red), and counterstained with DAPI (blue). The thickness of the corpus callosum is indicated with a white bar. Scale bar: 500 microns. CC: corpus callosum; LV: lateral ventricle. C-D. Hippocampal sections stained against Calbindin (green) and DKK3 (red) and counterstained with DAPI (blue). Scale bar: 250 microns. CA1-CA3: hippocampal regions, DG: dentate gyrus. **E-F.** Cortical sections stained with TBR1 (green) and CTIP2 (magenta), and counterstained with DAPI (blue). Scale bar: 100 microns. **G-H:** Quantifications of CTIP2+ and TBR1+ cells/area, respectively in control and Notch1cKO samples. **I-J.** Quantification of ratio of EdU+ cells colabeled with either CTIP2 or TBR1. For all quantifications (G-J), p-values were obtained using Student's T-tests. Scale bars: 500µm A-B, 250µm C-D, 100µm E-F.

Development • Supplementary information

Supplementary Figure 7

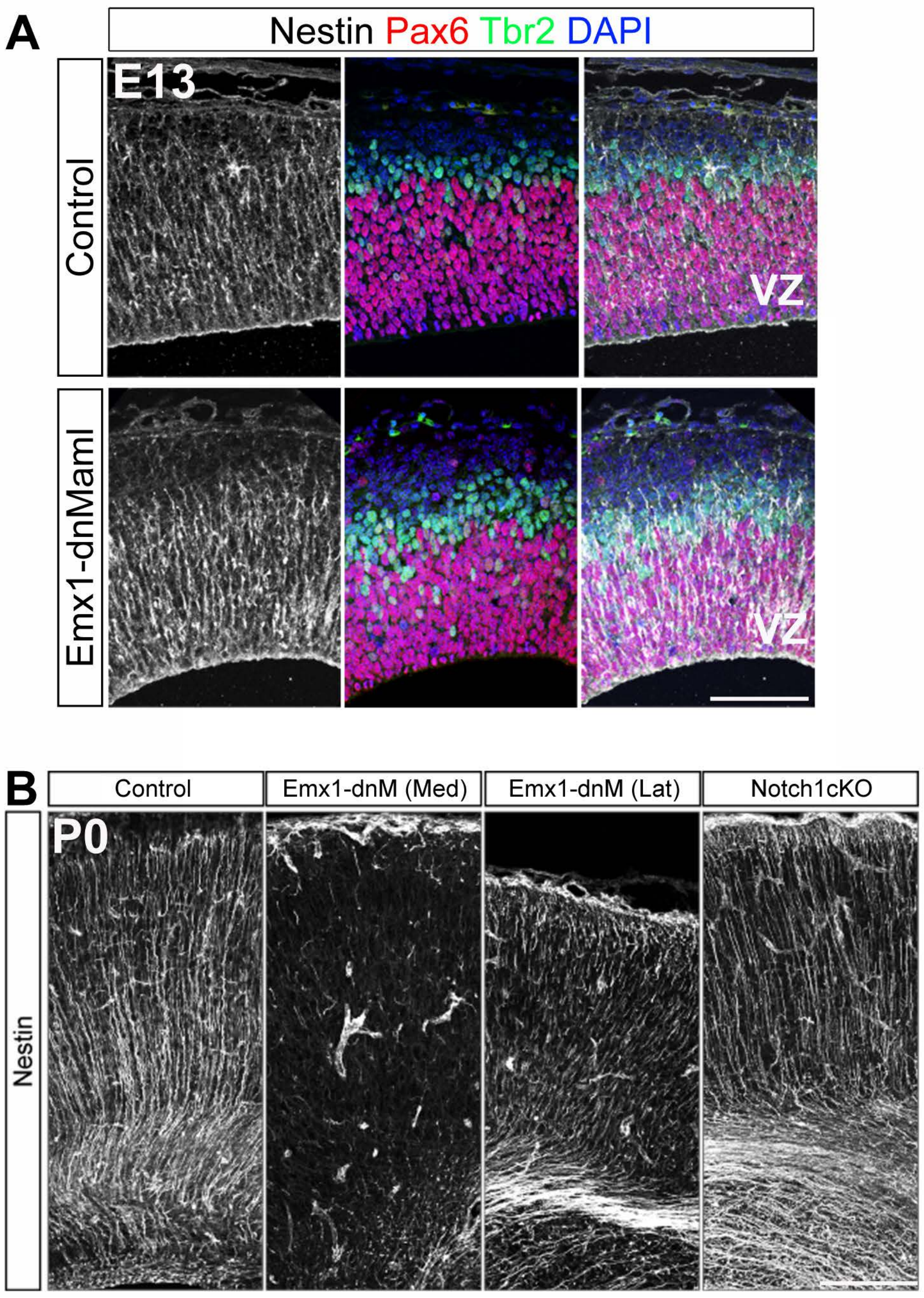

Fig. S7. Radial glia in Emx1-dnMAML and Emx1-Notch1cKO

A. Nestin (white), PAX6 (red) and TBR2 (green) antibody labeling of control and Emx1-dnMAML cortices were counterstained with DAPI (blue). **B.** Nestin (white) immunolabeling of cortices from control samples, Emx1-dnMAML, and Emx1-Notch1cKO. Note the difference between the medial (dnMAML-med) and lateral aspects (dnMAML-lat) of the cortices upon dnMAML upregulation. VZ: ventricular zone. Scale bar: 100μm.

Development • Supplementary information

Supplementary Figure 8

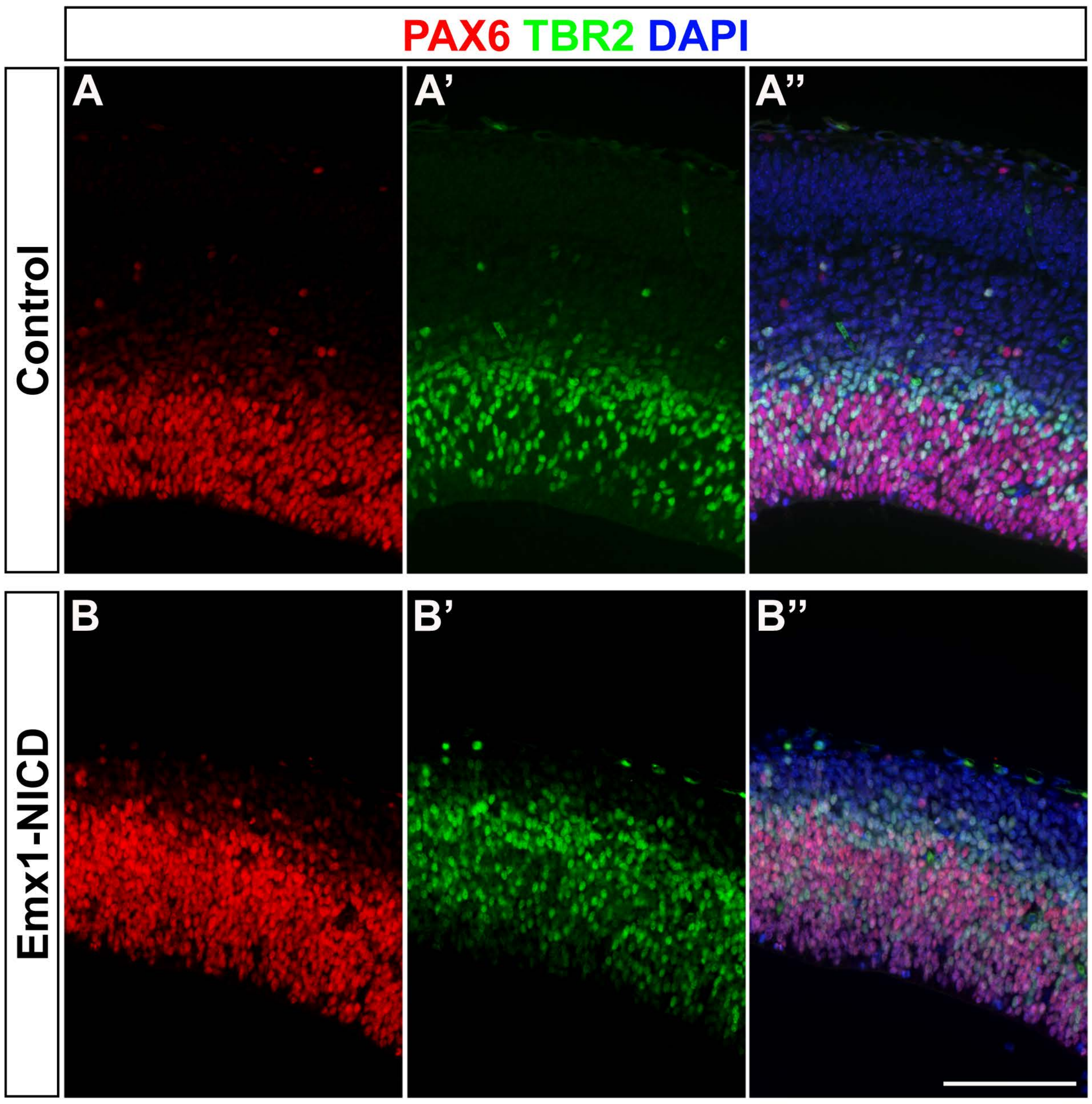

Fig. S8. TBR2+ intermediate progenitors at E15.5

PAX6 (red), TBR2 (green) and DAPI (blue) in Control (A-A'') and Emx1-NICD (B-B'') cortices at E15.5. Scale bar: 100μm.

Development • Supplementary information

Supplementary Figure 9

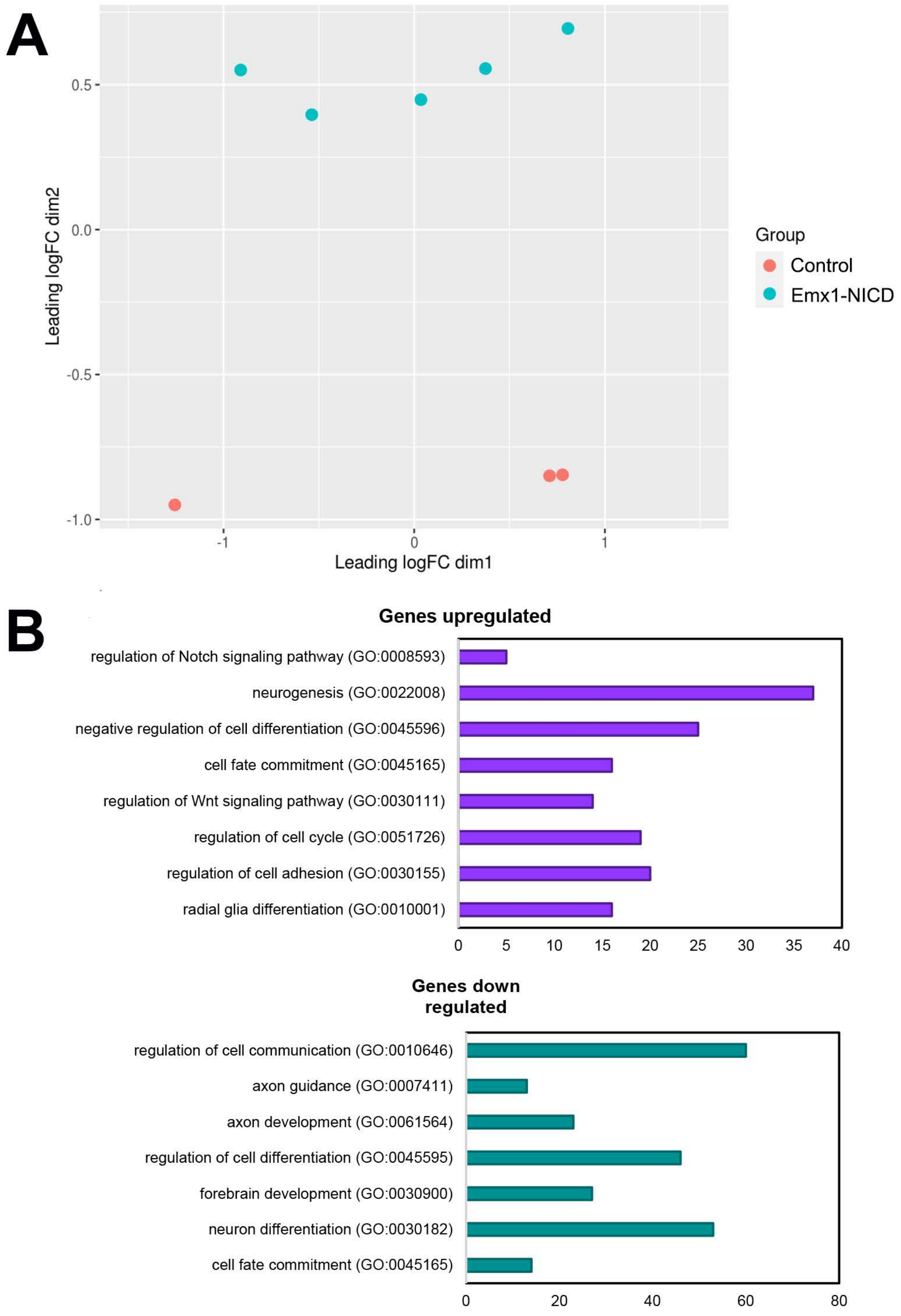

Fig. S9. RNA sequencing

A. Multidimensional scaling analysis (MDS) showing control samples (orange) and Emx1-NICD samples (teal). **B.** Gene Ontology (GO) analyses using PANTHER Classification System.

Development • Supplementary information

Supplementary Figure 10

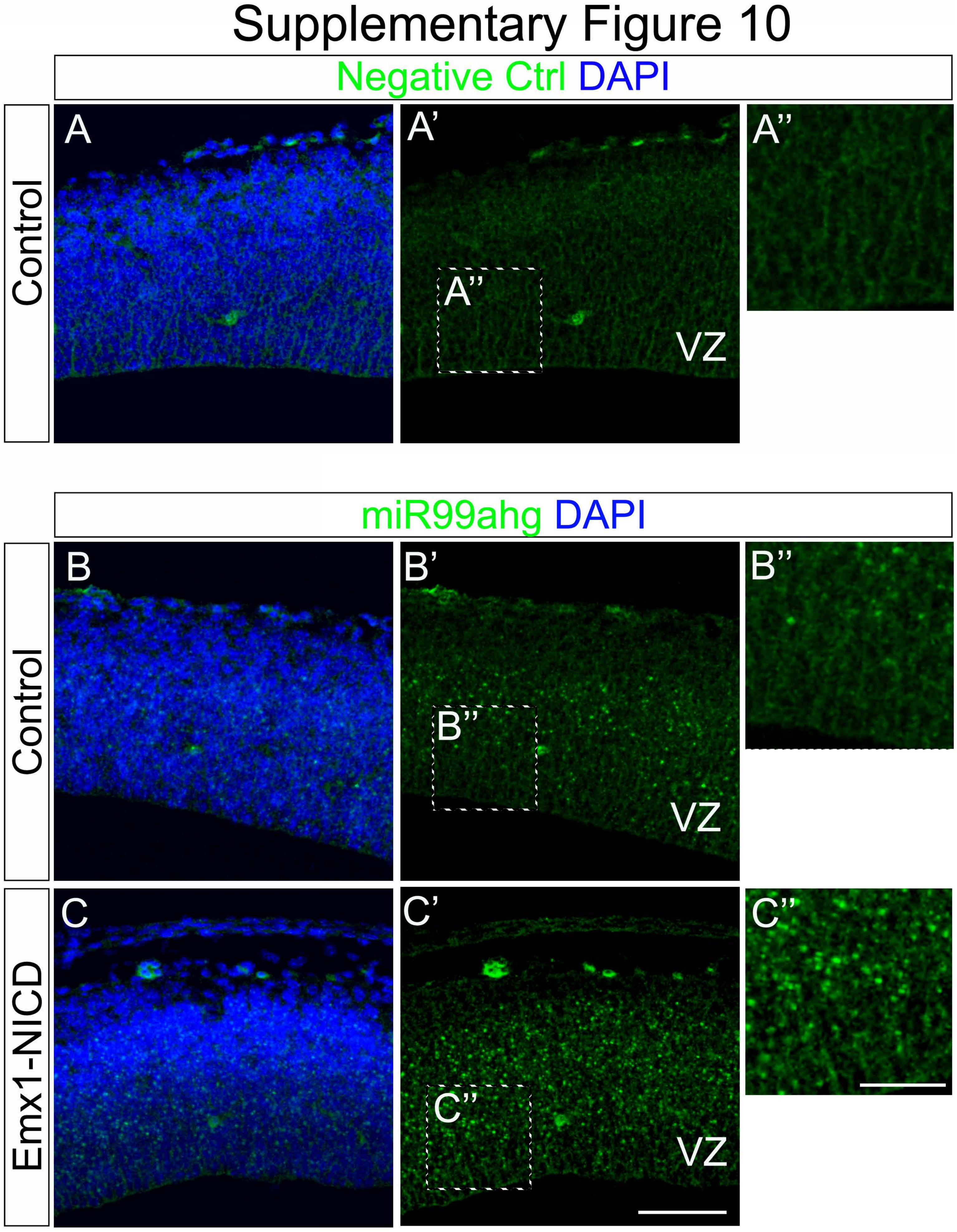

Fig. S10. Validation of RNA sequencing using RNAscope

A-A''. Negative control probe (green) counterstained with DAPI (blue). B-C'' miR99ahg probe signal is shown in green in control (B-B'') and Emx1-NICD mice (C-C''). These samples have also been counterstained with DAPI (blue). Scale bar: 100μm in C' and 50μm in C''.

Table S1. RNA-seq Differential Expression Normalized counts of Emx1-NICD vs Control samples.

Click here to download Table S1

Table S2. microRNA-seq Differential Expression Normalized counts of Emx1-NICD vs Control samples.

Click here to download Table S2

Development • Supplementary information

Table S3. Antibody details

Antibody	Source	Catalog	Lot	Concentration
Anti-BrdU (Mouse)	ThermoFisher	B35128	2125239	1:100
Anti-Calbindin (Mouse)	Millipore Sigma	C9848-100UL	079M4794V	1:200
Anti-Calretinin (Goat)	Swant	CG1	N/A	1:50
Anti-Cleaved Caspase-3 (Rabbit)	Cell Signaling Technologies	9664S	22	1:200
Anti-CTIP2 (Rat)	Abcam	ab18465	GR3462350-1	1:500
Anti-CUX1 (Rabbit)	Proteintech	11733-1-AP	00098340	1:1000
Anti-CUX1 (Rabbit)	Santa Cruz Biotechnologies	sc-13024, discontinued	CDP M-222X	1:50
Anit-DKK3 (Rabbit)	Sino Biological	50247-RP02	HB04MA1301-B	1:200
Anti-EOMES (TBR2) (Rat)	ThermoFisher	14-4875-82	2504948	1:200
Anti-FOXG1 (Rabbit)	Abcam	ab196868	GR3242662-8	1:100
Anti-GFAP (Mouse)	Antibodies Incorporated	73-240	455-8JD-45C	1:100
Anti-L1 (Rat)	Millipore Sigma	MAB5272	3663105	1:500
Anti-MSX1 (Goat)	R&D Systems	AF5045	CALG0119111	1:100
Anti-Nestin (Mouse)	BD Biosciences	556309	6084618	1:100
Anti-PAX6 (Rabbit)	BioLegend	901301	B386304	1:200
Anti-PH3 (Rabbit)	Millipore Sigma	06-570	3795233	1:100
Anti-Reelin (Mouse)	Millipore Sigma	MAB5364	3439219	1:100
Anti-RFP (Rabbit)	Novus Biologicals	NBP2-25157	71615	1:1000
Anti-RFP (Goat)	Origene	AB0040-200	N/A	1:1000
Anti-SATB2 (Mouse)	Abcam	51502	N/A	1:200
Anti-TBR1 (Rabbit)	Invitrogen	PA5-34582	WG3332981A	1:1000
Anti-TBR2 (Rabbit)	Abcam	ab23345	GR3230866-2	1:200
Anti-β-III-Tubulin (TUJ1) (Mouse)	BioLegend	801201	B264428	1:500

Development • Supplementary information
