# Supplementary material for: Notch directs telencephalic development and controls neocortical neuron fate determination by regulating microRNA levels
Source: Development. 2023 Jun 5;150(11):dev201408. doi: 10.1242/dev.201408 (PMC10309580; doi:10.1242/dev.201408)
Supplement: Supplementary file 1 [file develop-150-201408_review_history.pdf]

Notch directs telencephalic development and controls neocortical neuron fate determination by regulating microRNA levels

Jisoo S. Han, Elizabeth Fishman-Williams, Steven C. Decker, Keiko Hino, Raenier V. Reyes, Nadean L. Brown, Sergi Simó and Anna La Torre
DOI: 10.1242/dev.201408

Editor: Debra Silver

Review timeline

Original submission:	27 October 2022
Editorial decision:	6 December 2022
First revision received:	3 April 2023
Editorial decision:	24 April 2023
Second revision received:	24 April 2023
Accepted:	28 April 2023

Original submission

First decision letter

MS ID#: DEVELOP/2022/201408

MS TITLE: Notch directs telencephalic development and neuron fate determination by regulating miRNA levels

AUTHORS: Jisoo S. Han, Elizabeth Fishman-Williams, Steven C. Decker, Keiko Hino, Raenier V. Reyes, Nadean L. Brown, Sergi Simo, and Anna La Torre

I have now received all the referees' reports on the above manuscript, and have reached a decision. The referees' comments are appended below, or you can access them online: please go to BenchPress and click on the 'Manuscripts with Decisions' queue in the Author Area.

As you will see, the referees express considerable interest in your work, but have some significant criticisms and recommend a substantial revision of your manuscript before we can consider publication. Reviewer 1 indicates additional necessary experiments to increase the rigor of your study, particularly given prior findings regarding Notch signaling, and raises important points for discussion. Reviewer 2 raises important concerns regarding the transcriptomic data including methodology, public databases, and validation.

If you are able to revise the manuscript along the lines suggested, will be happy receive a revised version of the manuscript. Your revised paper will be re-reviewed by one or more of the original referees, and acceptance of your manuscript will depend on your addressing satisfactorily the reviewers' major concerns. Please also note that Development will normally permit only one round of major revision. If it would be helpful, you are welcome to contact us to discuss your revision in greater detail. Please send us a point-by-point response indicating your plans for addressing the referee’s comments, and we will look over this and provide further guidance.

Please attend to all of the reviewers' comments and ensure that you clearly highlight all changes made in the revised manuscript. Please avoid using 'Tracked changes' in Word files as these are lost in PDF conversion. I should be grateful if you would also provide a point-by-point response detailing

© 2023. Published by The Company of Biologists under the terms of the Creative Commons Attribution License (https://creativecommons.org/licenses/by/4.0/).

how you have dealt with the points raised by the reviewers in the 'Response to Reviewers' box. If you do not agree with any of their criticisms or suggestions please explain clearly why this is so.

Reviewer 1

Advance summary and potential significance to field

In this article, Han et al. investigate Notch signaling function in the neocortex using three mouse lines, (1) a line expressing Notch intracellular domain (NICD), (2) a line expressing a dominant negative form of the Notch co-activator MAML1 and (3) a conditional knockout line of Notch1. All these lines rely on the Emx1-Cre allele to either separately activate the expression of NICD or MAML1, or to knockout Notch1. In these lines the authors report alterations of corpus callosum development, as well as reduced hippocampi, impaired production of Cajal Retzius neurons at the cortical hem, an imbalance in the production of lower- and upper-layer projection neurons, a loss of intermediate progenitors and impaired cell cycle dynamics in radial glia. Looking for a molecular mechanism, they performed bulk RNA- and miRNA-sequencing in a population of embryonic cortical cells enriched for progenitors. Based on these results and to test the importance of miRNAs downstream of Notch signaling, the authors attempted a rescue of CTIP2+ neuron phenotypes in NICD cortices, using in utero electroporation of vectors expressing sponges for candidate miRNAs. The quality of the data and the attention to details is excellent. While the approach is not novel and many other studies have investigated the role of Notch signaling during cortical development, I think that a lot of the findings of this paper will be of interest to the field of cortical development and notch signaling at large. However, I also think several key experiments are required to firm up the conclusions of this study.

Comments for the author

Major concerns
1) While I think overexpressing NICD and dnMAML1 are useful tools to understand Notch function, these approaches are very artificial in nature. Notch may have some function in post-mitotic neurons, but many previous studies demonstrate that it is mainly active at a high level in progenitors. Are the neuron defects downstream consequences of progenitor defects, or are they due to a forced expression of NICD or dnMAML1 in cells that would otherwise not express it? I am not convinced that the authors can really conclude either way based on their data and should (1) tone down their conclusions and (2) discuss this caveat more thoroughly in the discussion.

2) The authors analyzed progenitors at only one timepoint during cortical development which makes it hard to connect the progenitor data to the changes in neuron fate. I think analyses at a later stage are warranted. Moreover, why didn't the authors study progenitor defects in dnMAML1 animals? This would be very helpful to further strengthen their conclusions.

3) I am concerned about the cell cycle analysis performed in PAX6+ cells only. In the WT, it is known that PAX6 protein remains expressed in the early stage of differentiation and that it can be found together with TBR2 as cells transition to an intermediate progenitor fate for instance. Since intermediate progenitors have been shown to have drastically different cell cycle dynamics, results in the WT could be "contaminated" by the inclusion of cells that show PAX6 protein but are actually neurons or intermediate progenitors during early differentiation. Therefore, the authors must redo this analysis to take this into account. This could be achieved through quadruple staining of EdU, BrdU PAX6 and TBR2. This is possible, see PMID: 26748089.

4) The miRNA rescue experiments need to consider other outcome measures beyond CTIP2 expression. What about TBR1 expression or the distribution of IUEd cells across the cortical thickness?

5) A previous group used a very similar approach overexpressing NICD but with a different Cre driver (Nestin-Cre, PMID: 15081359). In this previous study the authors observed massive apoptosis due to p53 activation early during cortical development. In the present study, Han et al. did not explore the contribution of early apoptosis to the phenotypes they observed at all, nor did they

© 2023. Published by The Company of Biologists under the terms of the Creative Commons Attribution License (https://creativecommons.org/licenses/by/4.0/).

compare their findings to those of that previous paper. This must be investigated in their model and differences between the two models should be discussed.

Minor concerns

1) Using only Reelin to assess Cajal Retzius fate is a little weak, as cells could start expressing reelin because of an “artificial” fate caused by overexpression of NICD or dnMAML1. The authors should use additional markers of CR neurons.

2) Along those lines, it would be interesting to quantify projection neuron numbers co-expressing different markers to test for possible “confused” neuronal fates.

3) In the dnMAML animals it would be helpful to visualize the radial glia scaffold, which could be a contributor to the aberrant migration they observed.

4) Regarding the ventricle size phenotypes, the authors should consider an alternative hypothesis whereby there could be increased and decreased tangential expansion of the progenitor pools in NICD and dnMAML animals, respectively.

Reviewer 2

Advance summary and potential significance to field

Han et al., made dorsal telencephalon specific gain and loss of function mice of Notch signaling by using NICD and dnMAML floxed transgenic animals respectively. Observations were also made in the cKO of Notch 1 driven by Emx1-cre. Descriptions of the dorsal telencephalic phenotypes are detailed, and appropriate tests for determining the effects of genetic manipulations on the cortical neuronal fates are made. FlashTag combined with FACS sorting in these genetic models sounds good strategy to specifically find the regulators under the Notch signaling in the RG. They also tested potential contributions of miRNAs that are known to have functions in the neuronal fate determination similar to the Notch signaling. By rescuing a phenotype of NICD overexpression with the sponge constructs, they, for the first time, showed the contribution of these miRNAs as downstream miRNAs of the Notch signaling to cortical development.

Comments for the author

My major concern is the lack of information about their transcriptome analysis making the readers and reviewers difficult to evaluate the reproducibility and rigor level of the study. Although known downstream targets are screened as expected, the presentation of the transcriptome data quality at the standard level in the field is required. Similarly, the tests of miRNA role are suboptimal to draw the conclusion; Does the miRNAs identified as downstream of the Notch signaling show corresponding gene expression pattern? Does overexpression of the miRNAs recapitulate the NICD phenotypes? It is unclear whether they used scrambled or mutated sponge constructs as a control. The amount of sponge constructs seems different between the experimental groups; zero sponge in control, 1.0 for single sponge, 1.5 for all three constructs together. The effects are significant in the experiments with higher number of the plasmids (= in the case of 1.5).

Major comments related to transcriptome data are found below:
1. Accessibility of the transcriptome data is not presented. Have the authors deposited the raw data to public domain?
2. Quality Check of the transcriptome is not provided.
3. Dry pipeline information is missing. Lack of information of methods used for processing from fastq file to count data. No information about DEG analysis algorithm or the cut-off setting of DEG.
4. No follow up wet validation by e.g. qRT-PCR or RNA in situ hybridization.
5. Sup Table 2: FDR adjusted p values are higher than that one imagines based on the volcano plot in the main figure.

© 2023. Published by The Company of Biologists under the terms of the Creative Commons Attribution License (https://creativecommons.org/licenses/by/4.0/).

A minor comment:

This manuscript describes the NICD conditional transgenic animal simply as NICD.
It may be confusing as the protein name also stands as NICD. For example.
The resulting mouse line, hereafter referred to as NICD, ….
Strikingly, NICD exhibits increased numbers of SATB2+ neurons compared to their littermate controls.

First revision

Author response to reviewers' comments

We would like to thank the reviewers for their insightful comments and interest in our manuscript. In the present resubmission, we have addressed all the issues raised by the reviewers, incorporated their suggestions, and provide additional data that had greatly improved our manuscript. A detailed point-by-point discussion of each reviewer's concerns is provided below:

Reviewer 1 Comments for the Author:

1) While I think overexpressing NICD and dnMAML1 are useful tools to understand Notch function, these approaches are very artificial in nature. Notch may have some function in post-mitotic neurons, but many previous studies demonstrate that it is mainly active at a high level in progenitors. Are the neuron defects downstream consequences of progenitor defects, or are they due to a forced expression of NICD or dnMAML1 in cells that would otherwise not express it? I am not convinced that the authors can really conclude either way based on their data and should (1) tone down their conclusions and (2) discuss this caveat more thoroughly in the discussion.

Thanks so much for the comment. Although our data correlate very well with Emx1-Cre;Rbpj and Emx1-Cre;Hes1/3/5 triple mutants, we agree with this concern and thus, we have decided to directly test whether the effects that we observed are a consequence of the "artifical" expression of these transgenes in postmitotic cells versus the effects at a progenitor level by expressing NICD and dnMAML only in postmitotic neurons using in utero electroporation of NeuroD2-Cre in $ROSA26^{loxP\text{-}STOP\text{-}loxP\text{-}NICD}$ or $^{ROSAloxP\text{-}STOP\text{-}loxP\text{-}dnMAML}$ mice. The proneural transcription factor NeuroD2 is expressed in the subventricular zone and cortical plate but not in the radial glia (PMID: 23303943, PMID: 31073041) and thus, this driver will not effect the apical progentiors.
In order to test the NeuroD2-Cre driver, we used the Double-UP construct (PMID: 32508591). This plasmid expresses mNeon (green) in the absence of Cre and mScarlett (red) after Cre- mediated recombination. As shown below (left panel), a co-electroporation *in utero* of Double-UP and NeuroD2-Cre led to the expression of mScarlett in the subventricular region but not in the ventricular progenitors, as predicted. For these proof-of-principle experiments, we electroporated mice at embryonic day 13.5 (E13.5) and collected the cortices 48-hours later (E15.5).
Next, we tested the effects of NICD and dnMAML overexpression in postmitotic cells by electroporating NeuroD2-Cre in $ROSA26^{loxP\text{-}STOP\text{-}loxP\text{-}NICD}$ or $ROSA^{loxP\text{-}STOP\text{-}loxP\text{-}dnMAML}$ mice at E13.5 also using Double-UP to monitor the cells undergoing recombination, and we dissected the brains at P0 for analyses. These samples were then immunolabaled with CTIP2 and counterstained with DAPI.

Interestingly, we did not observe the same phenotypes than when we used the Emx1-Cre driver. When NICD or dnMAML are expressed in postmitotic cells, many of these cells co-expressed CTIP2, as shown in below (right panel). Additionally, we did not observe obvious differences in position between the green (control) cells and the red (NICD-OE or dnMAML-OE) cells.

We believe these preliminary data strongly suggest that the phenotypes observed are initiated at a progenitor level, but since we cannot completely rule out some effects at a postmitotic level, we have also added some sentences in the manuscript to discuss this possibility, including the following:

© 2023. Published by The Company of Biologists under the terms of the Creative Commons Attribution License (https://creativecommons.org/licenses/by/4.0/).

"*Even though effects driven by the ectopic expression of NICD or dnMAML in post-mitotic neurons cannot be completely ruled out, our data support the idea that fate decisions are decided at the progenitor stage*"

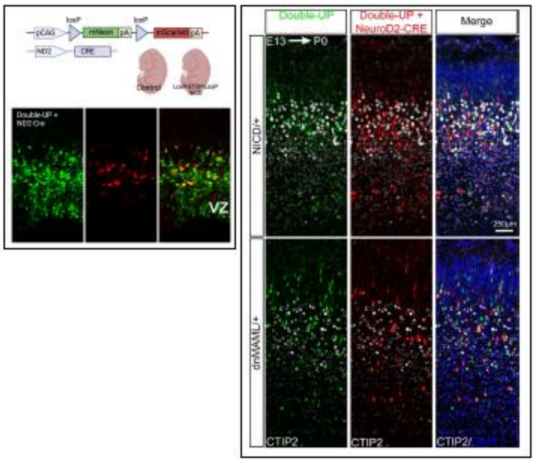

2) The authors analyzed progenitors at only one timepoint during cortical development which makes it hard to connect the progenitor data to the changes in neuron fate. I think analyses at a later stage are warranted. Moreover, why didn't the authors study progenitor defects in dnMAML1 animals? This would be very helpful to further strengthen their conclusions.

Unfortunately, at this time our Emx1-dnMAML animal colony is breeding poorly, thus too small to run new experiments in embryos to generate sufficient biologic replicates within the time frame for revision. We agree these data are interesting and worth pursuing once mouse breeding is restored, but we also don't think that the main conclusions of the paper would change significantly. We chose E13.5 because it is the age when CTIP2+ cells are born and these are the cells that exhibit the greatest differences between genotypes in our analyses. Additionally, E13.5 corresponds roughly to the middle of neurogenesis, allowing us to observe differences in both directions (precocious or delayed cell fates). Notably, when we looked at Emx1-dnMAML progenitors (Fig. 5), we observe oposite effects found for Emx1-NICD as was predicted to occur (lengthening cell cycle upon NICD overexpression vs shortening cell cycle in Emx1-dnMAML).

3) I am concerned about the cell cycle analysis performed in PAX6+ cells only.
In the WT, it is known that PAX6 protein remains expressed in the early stage of differentiation and that it can be found together with TBR2 as cells transition to an intermediate progenitor fate for instance. Since intermediate progenitors have been shown to have drastically different cell cycle dynamics, results in the WT could be "contaminated" by the inclusion of cells that show PAX6 protein but are actually neurons or intermediate progenitors during early differentiation. Therefore, the authors must redo this analysis to take this into account. This could be achieved through quadruple staining of EdU, BrdU, PAX6 and TBR2. This is possible, see PMID: 26748089.

Thank you so much for raising this important point. We agree that this is a concern and to address it, we attempted quadruple staining to distinguish the PAX6+ TBR2- from the PAX6+ TBR2+ cohorts. Unfortunately, none of the secondary antibodies conjugated with Alexa350 were reliable and the EdU Click-it detection kits are only available for Alexa Fluor 488, 555, 597 or 647 but not 350. Thus, we could not expand this experiment to simultaneously compare and quantify these subpopulations. Given this limitation, we performed PAX6 stainings in combination with TBR2 stainings to measure the proportion of PAX6 cells that are transitioning into intermediate progenitors or newly born postmitotic neuron fates (Figure below).

© 2023. Published by The Company of Biologists under the terms of the Creative Commons Attribution License (https://creativecommons.org/licenses/by/4.0/).

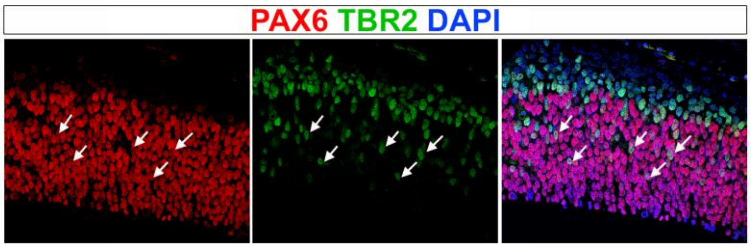

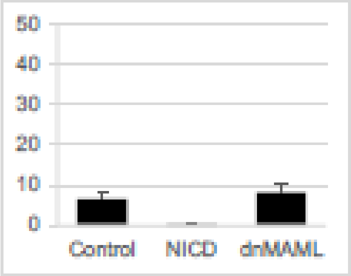

At E13.5, we detected only a minor overlap between the PAX6 and TBR2 populations, with 6.6 ± 1.75% of cells coexpressing PAX6 and TBR2 and thus, the majority of cells quantified (93.4%) were "true" RGs.

Alhtough there was statistical significant differences between control and Emx1-NICD samples, as we did not observe any TBR2+ cell at E13.5, there was no difference between controls and Emx1-dnMAML (p-value: 0.81). We do not believe that this "contamination" of <7% cells in controls and Emx1-dnMAML samples will skew the data in a significant way. Notably, it has been shown that TBR2+ intermediate progenitors have longer cell cycle length than PAX6+ TBR2- progenitors (PMID: 21224845) and thus, the comparison between controls and Emx-NICD mice in which we detect a significant lengthening of the cell cycle length but no TBR2+ cells are present may even be more significant than we report.

We then extended these analyses to E15.5 where we dected a more extensive double positive population (33% in controls and 41% in Emx1-NICD), and thus we have not included cell cycle quantifications in these conditions as the presence of PAX6+ TBR2+ cells could bias the overall results. However, these novel data have revealed that even though we do not observe TBR2+ intermediate progenitors at E13.5 in Emx1- NICD mice, TBR2+ intermediate progenitors are present at E15.5 and thus, the lack of TBR2+ cells at E13.5 could be interpreted as a delay.

In light of these new findings, we have modified the manuscript and added a new Supplementary figure 6 (see below). The text now reads as follows:

"In order to discriminate whether the lack of TBR2+ progenitors observed is a developmental delay or a permanent loss of intermediate progenitors, we extended these analyses to E15.5. Interestingly, at E15.5, we observed TBR2+ cells at similar ratios than the littermate controls (Supplementary Figure 7)."

© 2023. Published by The Company of Biologists under the terms of the Creative Commons Attribution License (https://creativecommons.org/licenses/by/4.0/).

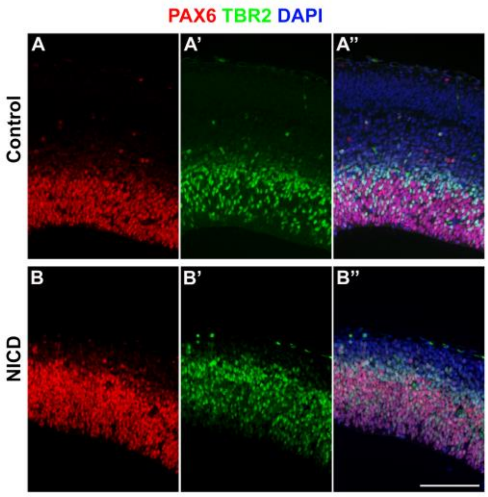

4) The miRNA rescue experiments need to consider other outcome measures beyond CTIP2 expression. What about TBR1 expression or the distribution of IUEd cells across the cortical thickness?

We quantified the distribution of IUEd cells in the rescue experiments. Control n=6, NICD n=4, let-7 only n=7, miR-125b only n=4, miR-99a/100 only n=4, all three sponges n=5.
As expected, we saw a shift in the distribution of IUEd cells in Emx1-NICD cortices compared to the control littermates as more cells located at the upper layers of the cortex. In control mice, bins 2-3 (upper-layers) contain 28.4% of all electroporated cells while we observed 53.8% of all IUEd cells in Emx1-NICD mice. Correspondingly, bins 5-6 contain 34.4% of cells in the control and only 12% of the electroporated cells upon NICD overexpression. Notably, the addition of the sponges either one-by-one or together reduced the number of cells in the upper parts of the cortex, while increasing the presence of cells in bins 4-5. However, in this case we do not observe a significant benefit from using all the sponges together vs. one by one. This may be due to the variable nature of the IUE experiments or because the molecular mechanisms regulating fate decisions and migration patterns are subtly different. A simplified version of this graph has now been included in Figure 7 (Figure 7H).

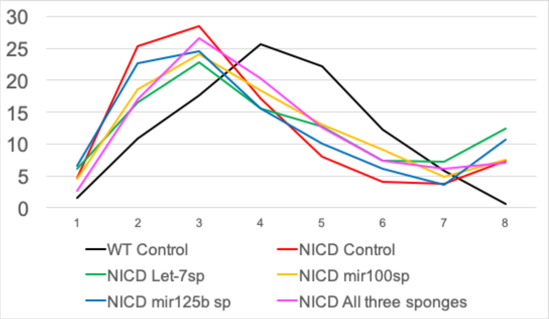

5) A previous group used a very similar approach overexpressing NICD but with a different Cre driver (Nestin-Cre, PMID: 15081359). In this previous study the authors observed massive apoptosis due to p53 activation early during cortical development. In the present study, Han et al. did not explore the contribution of early apoptosis to the phenotypes they observed at all, nor did they compare their findings to those of that previous paper. This must be investigated in their model and differences between the two models should be discussed.

Since the size of the telencephalic structures—especially the hipocampus—was smaller in our Emx1-NICD mice at postnatal stages, we tested if there was augmented apoptosis in these mice at P0 (Supplementary figure 3). To further address the possibility of earlier effects, we have now tested activated Caspase 3 and p53 in E13.5 samples (Figure below). We did not observe increased apoptosis in the Emx1-NICD mice with either marker. Additionally, as shown in Figure 2, at these

© 2023. Published by The Company of Biologists under the terms of the Creative Commons Attribution License (https://creativecommons.org/licenses/by/4.0/).

early stages, the cortical anlage is longer in Emx1-NICD samples compared to control littermates, but the thinkness of the cortex is not significantly different, suggesting that massive apoptosis has not happened earlier.

Since both Nestin and Emx1 are expressed in the radial glia from early time-points, we do not think the differences are due to expression patterns. Instead, the differences between these models could be attributed to dosage. A possible hypothesis is that Nestin-Cre perhaps drives expression of NICD to very high levels leading to apoptosis, while the Emx1-Cre driver leads to a more modest upregulation of NICD; but of course, this is only speculation.

Given the space limitations, we have not included these data in the manuscript.

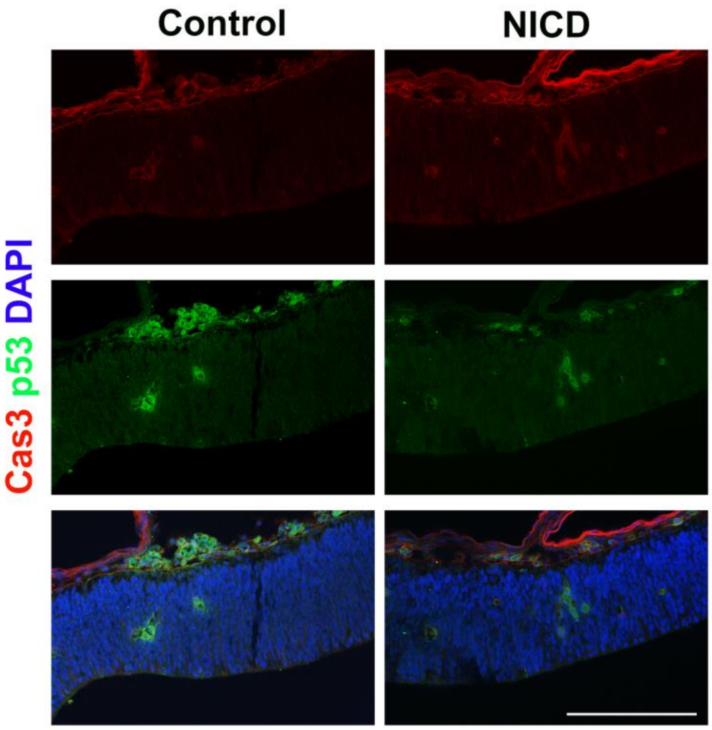

Minor concerns

1) Using only Reelin to assess Cajal Retzius fate is a little weak, as cells could start expressing reelin because of an “artificial” fate caused by overexpression of NICD or dnMAML1. The authors should use additional markers of CR neurons.

Thanks for the comment. We agree and thus, we used a combination of Reelin, Calretinin, and TBR1 to distinguish Cajal-Retzius cells. TBR1 is only expressed in Cajal-Retzius cells at early stages of development (E10.5-E14) but not later (PMID: 12644247). Consequently, in the cortex, we observed Reelin+ Calretinin+ TBR1+ Cajal-Retzius cells at E13.5 and Reelin+ Calretinin+ TBR1- Cajal-Retzius cells at E15.5 (Figure below, left panel). Interestingly, when we analyzed the Emx1-NICD hipocampal anlage at E13.5, we saw that the Cajal-Retzius cells located in the normal position (ie., within the hippocampal primordia) express all the markers, including TBR1, but the ectopic Reelin+ cells that we observed in the Choroid plexus region (white arrows in right panel) co-express Calretinin but not TBR1.

As suggested by this reviewer, these new data suggests that the Reelin+ cells ectopically located may not be “true” Cajal-Retzius cells or alternatively, these are Cajal-Retzius cells at a different developmental state. Thus, we have toned down the discussion regarding these cells: we call them now “Reelin+ cells” instead of “Cajal-Retzius cells” and the possibility of an aberrant expression of some markers in choroid plexus cells is discussed. The edited paragraph now reads: *"However, while the ChP is properly patterned and establishes a sharp boundary with the CH, we identified ectopic Reelin+ cells in the ChP region at E13.5 in our Emx1-NICD model. Notably, these*

© 2023. Published by The Company of Biologists under the terms of the Creative Commons Attribution License (https://creativecommons.org/licenses/by/4.0/).

ectopic cells express Reelin and Calretinin (both markers of Cajal-Retzius) but not TBR1, a marker normally detected in Cajal-Retzius cells at E13.5 but not expressed in these cells at later stages (Hevner et al. 2003). A previous study using lineage-tracing analysis of the prospective ChP region indicated that these progenitors sequentially give rise to Reelin+ Cajal-Retzius cells first and later to nonneural ChP epithelial fates (Imayoshi et al., 2008). Inactivation of Hes1, Hes3 and Hes5 genes led to an enhanced development of Cajal-Retzius cells at the expense of ChP cell fates, suggesting that Notch signaling regulates the progenitor transition from cells that produce Cajal-Retzius cells to progenitors that will generate nonneural ChP epithelium cell identities. However, around E12.5, the levels of HES1 and HES5 are downregulated after the ChP cell fate is specified (Imayoshi et al., 2008). The presence of ectopic Reelin+ cells in our data suggests that this later dowregulation of Notch signaling may be required to maintain the ChP fate and that ChP progenitors can transdifferentiate to neural fates or at least express some neural markers upon sustained HES1/5 activity. The ectopic Reelin+ cells observed could alternatively be the consequence of aberrant migratory patterns from the CH."

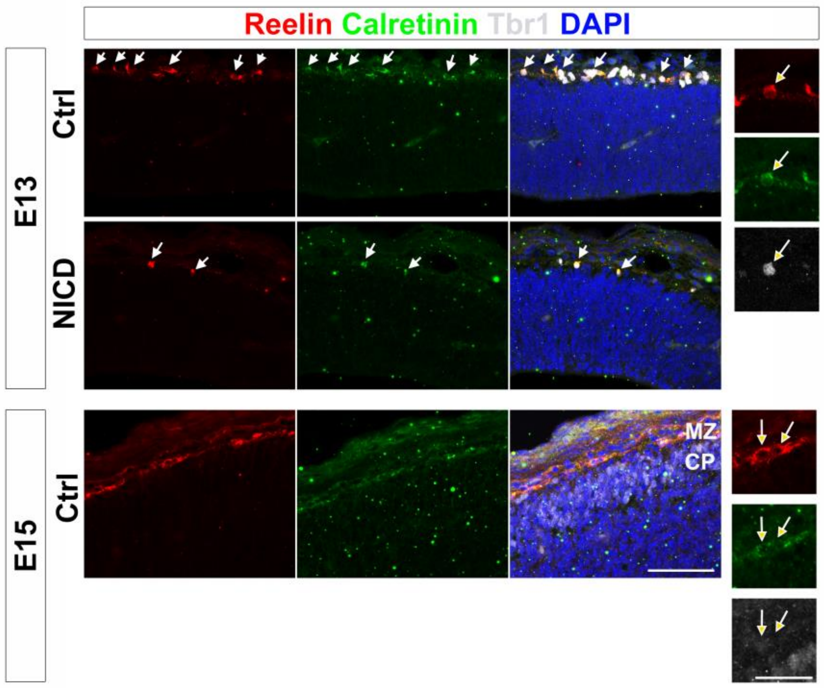

© 2023. Published by The Company of Biologists under the terms of the Creative Commons Attribution License (https://creativecommons.org/licenses/by/4.0/).

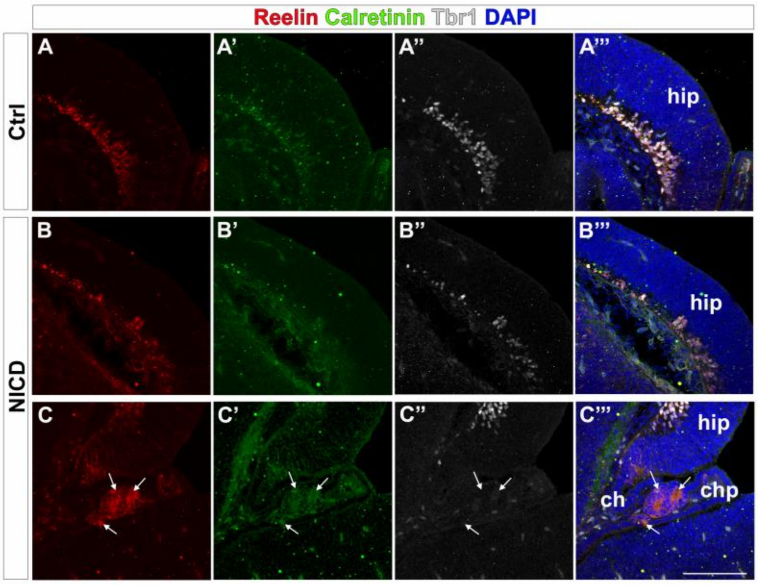

2) Along those lines, it would be interesting to quantify projection neuron numbers co-expressing different markers to test for possible "confused" neuronal fates.

During our previous analyses, we did not observed obvious changes in the patterns of expression of the cortical markers (i.e., we did not observe higher/lower colocalization rates between markers nor changes in the levels of expression), but in order to quantify this possibility we also quntified the percentages of CUX1/ CTIP2 double-positive cells in control and Emx1-NICD cortices at P10. As shown in the figure, we did not observe a significant difference between genotypes (p-value = 0.63, n=3/condition).

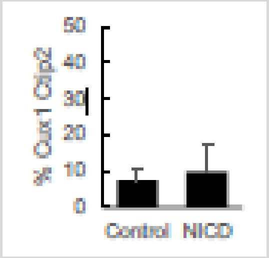

3) In the dnMAML animals it would be helpful to visualize the radial glia scaffold, which could be a contributor to the aberrant migration they observed.

Thank you for raising this important point. We agree that the radial glia may be a contributing factor of the aberrant migration as described for Emx1-RBPJ cKO mice (PMID: 32780108). In order to test this possibility, we labeled E13.5 and P0 cortices with Nestin to mark the radial glia. As shown in the new Supplementary figure 7 (see below), we do not observe changes in the organization of the radial glia. However, at P0 we observed that the cortex is depleted of these cells in the medial regions, but not in the lateral aspects. Since Emx1-Notch cKO cortices also display migration defects, albeit less severe than in Emx1-dnMAML samples, we also tested Nestin in Emx1-Notch cKO. Again, we did not observe apparent differences in the organization of the radial glia and in our models, the radial glia did not display the curved apearance and uneven distribution detected in Emx1-RBPJ cKO mice. However, RBPJ binds to chromatin in NICD-containing complexes that activate transcription but also in context-dependent co-repressor (Notch-independent) complexes that repress transcription and therefore, the RBPJ model may exhibit Notch-independent phenotypes that differ from our models.

© 2023. Published by The Company of Biologists under the terms of the Creative Commons Attribution License (https://creativecommons.org/licenses/by/4.0/).

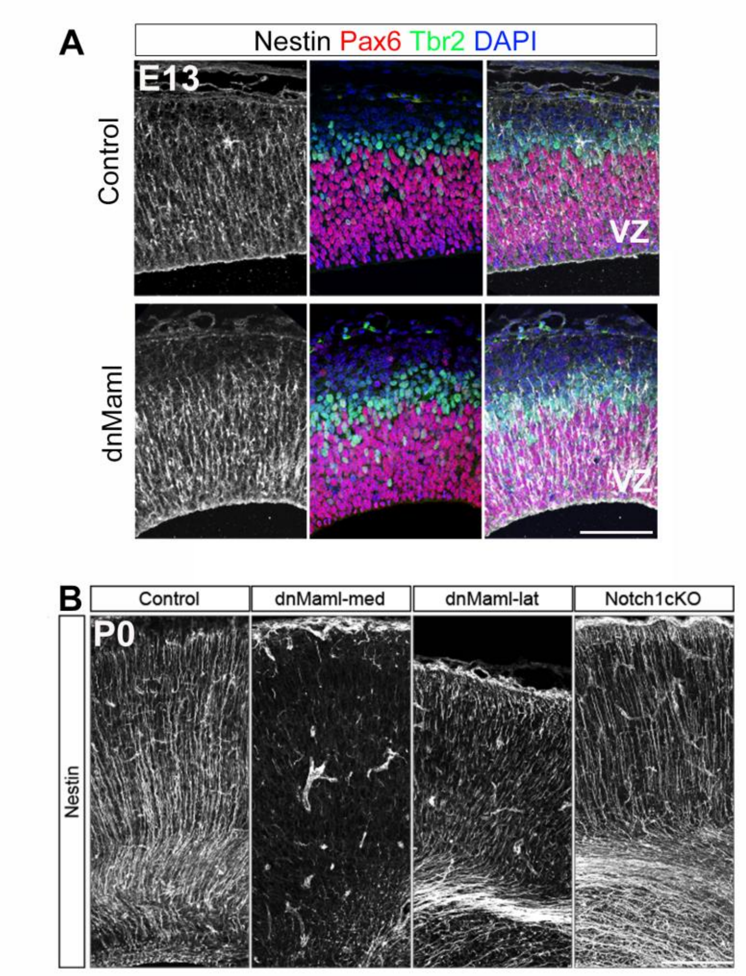

4) Regarding the ventricle size phenotypes, the authors should consider an alternative hypothesis whereby there could be increased and decreased tangential expansion of the progenitor pools in NICD and dnMAML animals, respectively.

Thanks for the suggestion. We have added this possibility as a discussion point.

Reviewer 2 Comments for the Author:

My major concern is the lack of information about their transcriptome analysis, making the readers and reviewers difficult to evaluate the reproducibility and rigor level of the study. Although known downstream targets are screened as expected, the presentation of the transcriptome data quality at the standard level in the field is required.

Major comments related to transcriptome data are found below:
1.Accessibility of the transcriptome data is not presented. Have the authors deposited the raw data to public domain?

2.Quality Check of the transcriptome is not provided.
3. Dry pipeline information is missing. Lack of information of methods used for processing from fastq file to count data. No information about DEG analysis algorithm or the cut-off setting of DEG.
4.No follow up wet validation by e.g. qRT-PCR or RNA in situ hybridization.
5.Sup Table 2: FDR adjusted p values are higher than that one imagines based on the volcano plot in the main figure.

Thank you so much for these comments. We apologize for our oversight in not including these on

© 2023. Published by The Company of Biologists under the terms of the Creative Commons Attribution License (https://creativecommons.org/licenses/by/4.0/).

the first version and as suggested, we have now made available all the information about quality controls, sequencing pipeline as well as all the raw data. As now indicated in the manuscript, all these information is available on the Dryad Data Repository hosted by UC Davis:

https://datadryad.org/stash/share/zlQ9xdEeXaL_r1m37zAuw5VtmSdslZeKELu4J38S2MU

As quality control we used MultiQC (v1.10.dev0) to assess percentage of alignment, aligment scores, gene counts, etc. We also made public our processiging pipelines and other standard quality control measures (fragment reduction, basepair reduction, hts stats, read lengths, uniformity of distribution, quality by cycle, etc…).

For mRNA sequencing: Adapter trimming, quality trimming of the ends of reads, filtering of sequences less than 50 bases, and removal of phiX sequences were conducted using HTStream, version 1.3.3 [1]. Removal of PCR duplicates identified by unique molecular indices (UMI) was conducted using UMI-tools, version 1.0.1 [2]. Reads were aligned to GRCm39 using STAR, version 2.7.3a [3]. Read counts for each gene were obtained using HTSeq, version 0.12.3 [4].

Differential expression analysis was conducted using the limma-voom Bioconductor pipeline [5]. Prior to analysis, genes with fewer than 5 counts per million reads in all samples were filtered prior to analysis, leaving 12,556 genes. Differential expression was defined as a Benjamini- Hochberg [6] adjusted p-value less than 0.05.

For miRNA sequencing: Adapter trimming and filtering of reads with fewer than 14 bases or more than 34 bases was performed using a custom Python script. Removal of phiX sequences was conducted using HTStream, version 1.3.3. Reads were aligned to GRCm39 using STAR, version 2.7.3a, using settings for miRNASeq recommended by the ENCODE project [7,8]. Feature counts were obtained using the featureCounts tool from Subread, version 1.6.3 [9], using settings that allowed multimappers.

Differential expression analysis was conducted using the limma-voom Bioconductor pipeline. Prior to analysis, miRNAs expressed in fewer than 3 samples were filtered, leaving 779 miRNAs. Differential expression was defined as a Benjamini-Hochberg adjusted p-value less than 0.05.

We have also performed wet validation by *in situ* (see the next point below).

References

1. https://s4hts.github.io/HTStream/
2. Smith T, Heger A, Sudbery I. UMI-tools: modeling sequencing errors in Unique Molecular Identifiers to improve quantification accuracy. Genome Res. 2017 Mar;27(3):491-499. doi: 10.1101/gr.209601.116. Epub 2017 Jan 18. PMID: 28100584; PMCID: PMC5340976.
3. Dobin A, Davis CA, Schlesinger F, Drenkow J, Zaleski C, Jha S, Batut P, Chaisson M, Gingeras TR. STAR: ultrafast universal RNA-seq aligner. Bioinformatics. 2013 Jan 1;29(1):15-21. doi: 10.1093/bioinformatics/bts635. Epub 2012 Oct 25. PMID: 23104886; PMCID: PMC3530905.
4. Anders S, Pyl PT, Huber W. HTSeq--a Python framework to work with high-throughput sequencing data. Bioinformatics. 2015 Jan 15;31(2):166-9. doi: 10.1093/bioinformatics/btu638. Epub 2014 Sep 25. PMID: 25260700; PMCID: PMC4287950.
5. Matthew E. Ritchie, Belinda Phipson, Di Wu, Yifang Hu, Charity W. Law, Wei Shi, Gordon K. Smyth, limma powers differential expression analyses for RNA-sequencing and microarray studies, Nucleic Acids Research, Volume 43, Issue 7, 20 April 2015, Page e47, https://doi.org/10.1093/nar/gkv007
6. Benjamini, Y., and Hochberg, Y. (1995). Controlling the false discovery rate: a practical and powerful approach to multiple testing. Journal of the Royal Statistical Society Series B, 57, 289--300.
7. https://www.encodeproject.org/
8. https://www.encodeproject.org/documents/b4ec4567-ac4e-4812-b2bd-e1d2df746966/@@download/attachment/ENCODE_miRNA-seq_STAR_parameters_v2.pdf
9. Liao Y, Smyth GK and Shi W. featureCounts: an efficient general-purpose program for assigning sequence reads to genomic features. Bioinformatics, 30(7):923-30, 201

© 2023. Published by The Company of Biologists under the terms of the Creative Commons Attribution License (https://creativecommons.org/licenses/by/4.0/).

Similarly, the tests of miRNA role are suboptimal to draw the conclusion; Does the miRNAs identified as downstream of the Notch signaling show corresponding gene expression pattern? Does overexpression of the miRNAs recapitulate the NICD phenotypes?

-In order to validate the RNAseq and miRNAseq data and assess the expression pattern of the miRNA clusters, we performed RNAscope *in situ* hybridization using a probe against miR99ahg. As shown below, there is an obvious increase in miR99ahg+ puncta in Emx1-NICD cortices compared to the control samples. We included this data as new Supplementary figure 9 (see below)

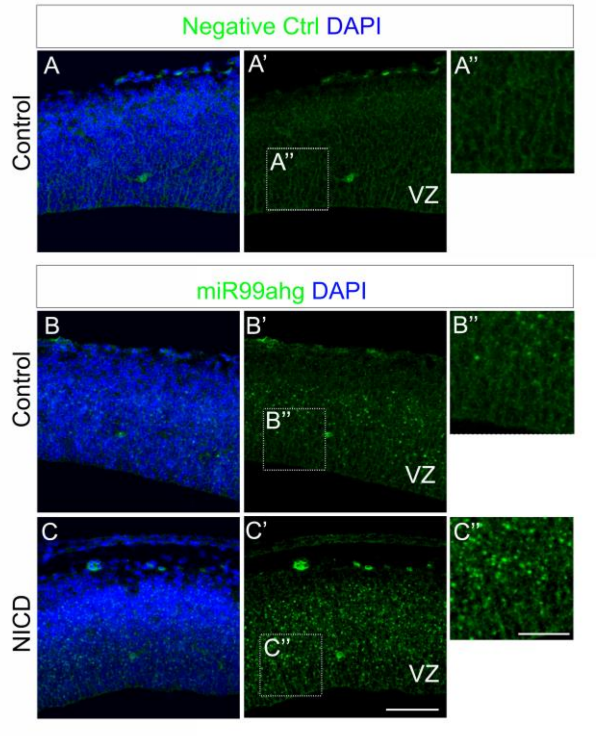

Similarly, the overexpression of these miRNAs in wild type E13.5 mice by in utero electroporation leads to the recapitulation of the Emx1-NICD phenotype, as shown below. In Fig 7A of the manuscript, control electroporations show that about 20% of the cells electroporated at E13.5 become CTIP2+ and they have a quite dispersed distribution thorougout the cortical plate (Fig. 7H). In contrast, the overexepression of let-7, miR-125b and miR-99a/100 together result in almost no colocalization (<3%) between the electroporated cells and CTIP2 and all the cells are now located in the upper layers, above the CTIP2+ cells (see figure below).
Notably, our experiments and also published data indicate that let-7 by itself is sufficient to induce these changes (PMID: 31080058) as overexpression of let-7 at E13.5 leads to a shift in the electroporated cells to generate upper-layer neurons.

© 2023. Published by The Company of Biologists under the terms of the Creative Commons Attribution License (https://creativecommons.org/licenses/by/4.0/).

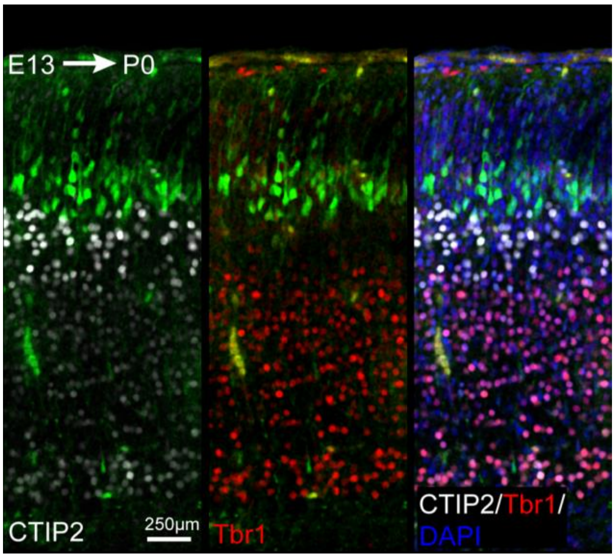

It is unclear whether they used scrambled or mutated sponge constructs as a control. The amount of sponge constructs seems different between the experimental groups; zero sponge in control, 1.0 for singlesponge, 1.5 for all three constructs together. The effects are significant in the experiments with higher number of the plasmids (= in the case of 1.5).

In cases where more than one sponge was electroporated, we used less of each sponge for technical reasons as we always make sure to keep the electroporation volumes consistent between conditions (1ul/electroporation). Additionally, the total amount of DNA was not drastically different between conditions (ranging from 1 to 3.5 ug). We and others routinely electroporate up to 5 different plasmids and up to 6ug of DNA (i.e., PMID: 32873638) without affecting fate, cell position or cell death in control conditions and thus, we do not believe that the number of plasmids is a factor in the results presented. As indicated, electroporation controls are empty vectors.

Additionally, our results show that using less of each sponge in the combinatorial approach was more efficient at rescuing the fate phenotypes, than higher concentrations of individual sponges. This supports our idea of synergystic effects among these miRNAs. We and others have published similar findings in other paradigms where a combination of miRNAs was sufficient and necessary to shift fate choices but individual miRNAs only had modest effects (i.e, PMID: 23754433).

A minor comment:
This manuscript describes the NICD conditional transgenic animal simply as NICD. It may be confusing as the protein name also stands as NICD. For example.
The resulting mouse line, hereafter referred to as NICD, ….
Strikingly, NICD exhibits increased numbers of SATB2+ neurons compared to their littermate controls.

Thank you for bringing up this point. For clarification, we have changed the name of the mutants analyzed from NICD to Emx1-NICD and from dnMAML to Emx1-dnMAML.

Second decision letter

MS ID#: DEVELOP/2022/201408

MS TITLE: Notch directs telencephalic development and neuron fate determination by regulating miRNA levels

AUTHORS: Jisoo S Han, Elizabeth Fishman-Williams, Steven C Decker, Keiko Hino, Raenier V Reyes, Nadean L Brown, Sergi Simo, and Anna La Torre

© 2023. Published by The Company of Biologists under the terms of the Creative Commons Attribution License (https://creativecommons.org/licenses/by/4.0/).

I have now received all the referees reports on the above manuscript, and have reached a decision. The referees' comments are appended below, or you can access them online: please go to BenchPress and click on the 'Manuscripts with Decisions' queue in the Author Area.

The overall evaluation is positive and we would like to publish a revised manuscript in Development, provided that the referees' comments can be satisfactorily addressed. Please attend to all of the reviewers' comments in your revised manuscript and detail them in your point-by-point response. If you do not agree with any of their criticisms or suggestions explain clearly why this is so. If it would be helpful, you are welcome to contact us to discuss your revision in greater detail. Please send us a point-by-point response indicating your plans for addressing the referee’s comments, and we will look over this and provide further guidance.

Reviewer 1

Advance summary and potential significance to field

I think this paper provides important data for the Notch and cortical development fields.

Comments for the author

The authors have performed additional experiments and provide new data to solidify their findings and conclusions.

Reviewer 2

Advance summary and potential significance to field

It stays the same as the initial submission.

Comments for the author

Most of the criticisms were well addressed except data presentation that is essential for reproducibility. The QC and processed data appear to be deposited to UC Davis site, but a file that contains fastq, QC and count data is 4.5 GB, and I kept having errors in downloading. I think people have different level of interests in these three datasets. In my case, I just want to check QC (probably it's less than 900kb) which is not available in original manuscript. But it requires me to download all of 4.5GB data which does not make sense. So, I am not able to evaluate data quality of the sequencing in this paper. In addition, if you have custom python code, I think the standard is that the data accessibility statement need to mention the availability.

Second revision

Author response to reviewers' comments

Regarding Reviewer #2 comment, it is true that when using the temporary link provided in our Response to Reviewers document to access the sequencing data hosted in Dryad, the only option was to download the complete dataset, adding up to several gigabits. I am glad to report that since April 5th, 2023, our Dryad submission was approved a readily available to the scientific community. Using the permalink provided in our manuscript, now the reviewer, and any reader, will be able to download single or multiple files, including the raw fastq files and the quality controls run on our

© 2023. Published by The Company of Biologists under the terms of the Creative Commons Attribution License (https://creativecommons.org/licenses/by/4.0/).

datasets. Furthermore, we added a sentence in our Data Accessibility statement indicating that any custom code to process sequencing data is available upon request.

Third decision letter

MS ID#: DEVELOP/2022/201408

MS TITLE: Notch directs telencephalic development and neuron fate determination by regulating miRNA levels

AUTHORS: Jisoo S Han, Elizabeth Fishman-Williams, Steven C Decker, Keiko Hino, Raenier V Reyes, Nadean L Brown, Sergi Simo, and Anna La Torre
ARTICLE TYPE: Research Article

I am happy to tell you that your manuscript has been accepted for publication in Development, pending our standard ethics checks.

© 2023. Published by The Company of Biologists under the terms of the Creative Commons Attribution License (https://creativecommons.org/licenses/by/4.0/).
